# Nuclear Magnetic Resonance-Based Metabolomics Approach to Evaluate the Prevention Effect of *Camellia nitidissima Chi* on Colitis-Associated Carcinogenesis

**DOI:** 10.3389/fphar.2017.00447

**Published:** 2017-07-11

**Authors:** Ming-Hui Li, Hong-Zhi Du, Gui-Ju Kong, Li-Bao Liu, Xin-Xin Li, Sen-Sen Lin, Ai-Qun Jia, Sheng-Tao Yuan, Li Sun, Jun-Song Wang

**Affiliations:** ^1^Center for Molecular Metabolism, Nanjing University of Science and Technology Nanjing, China; ^2^Jiangsu Key Laboratory of Drug Screening and Jiangsu Center for Pharmacodynamics Research and Evaluation, China Pharmaceutical University Nanjing, China; ^3^Department of Cardiothoracic Surgery, The Third Affiliated Hospital, Sun Yat-Sen University Guangzhou, China; ^4^Tasly Research Institute, Tianjin Tasly Holding Group Co. Ltd. Tianjin, China; ^5^School of Environmental and Biological Engineering, Nanjing University of Science and Technology Nanjing, China

**Keywords:** *Camellia nitidissima Chi*, AOM/DSS, colorectal carcinogenesis, metabolomics, OPLS-DA, correlation network analysis

## Abstract

Colorectal cancer (CRC) is one of the most common malignant tumors worldwide, occurring in the colon or rectum portion of large intestine. With marked antioxidant, anti-inflammation and anti-tumor activities, *Camellia nitidissima Chi* has been used as an effective treatment of cancer. The azoxymethane/dextran sodium sulfate (AOM/DSS) induced CRC mice model was established and the prevention effect of *C. nitidissima Chi* extracts on the evolving of CRC was evaluated by examination of neoplastic lesions, histopathological inspection, serum biochemistry analysis, combined with nuclear magnetic resonance (NMR)-based metabolomics and correlation network analysis. *C. nitidissima Chi* extracts could significantly inhibit AOM/DSS induced CRC, relieve the colonic pathology of inflammation and ameliorate the serum biochemistry, and could significantly reverse the disturbed metabolic profiling toward the normal state. Moreover, the butanol fraction showed a better efficacy than the water-soluble fraction of *C. nitidissima Chi*. Further development of *C. nitidissima Chi* extracts as a potent CRC inhibitor was warranted.

## Introduction

Colorectal cancer (CRC) is one of the most common malignant tumors worldwide, occurring in the colon or rectum portion of large intestine in the gastrointestinal tract. In China, CRC is the fourth and fifth most common diagnosed cancer among women and men respectively, and the fifth leading causes of cancer death (Chen et al., 2016). More seriously, CRC patients tends to be younger and younger due to the increasing life pressure, unhealthy living habit, polluted environment, and so on. However, it has been estimated that nearly 60% of cancer deaths can be avoided by reducing exposure to modifiable risk factors (Wang et al., 2012). The three largest factors contributing to CRC in China are ulcerative colitis, familial adenomatous polyposis, and hereditary non-polyposis colon cancer syndrome. Patients with ulcerative colitis were considered to be of high risk of CRC, the pathogenesis of which was believed to be evolved as a step-wise progression from inflamed and hyperplastic epithelia to flat dysplasia, and finally to adenocarcinoma (Thorsteinsdottir et al., 2011). While the reactive oxygen species (ROS), which can cause oxidative DNA damage, is thought to be one of the most causes for ulcerative colitis and CRC.

*Camellia nitidissima Chi* is a world-famous economic and ornamental evergreen shrub with golden-yellow flowers, growing mainly in Guangxi province of China (Yang et al., 2008). It has been classified as one of the rarest plants, known as “flora panda” and “camellia queen” (Yang et al., 2008). It contains a variety of physiologically active ingredients, such as tea polysaccharides, polyphenols, flavonoids, and tea saponins, which has been reported that its leaves, flowers, and seed oils can be of value in food and Chinese traditional medicine, playing an important role in health care for human beings to treat sore throat, diarrhea, high blood pressure, blood in the stool, irregular menstruation, and for cancer prevention (Liang, 1993). Clinical findings showed that the plants could inhibit the transplanted cancer, lower blood pressure, lower blood lipid, lower cholesterol, and prevent atherosclerosis (Huang et al., 2009; Qi et al., 2016). With significant antioxidant, anti-inflammation and anti-tumor activity, *C. nitidissima Chi* has been applied as an effective treatment for cancer. However, few of these efficacies have been confirmed by *in vivo* experiments, owing to the complexity of its components and associated complicated mechanisms.

Metabolomics, as a systemic perspective of endogenous metabolic profiles in biological samples, reflects the fluctuations of small-molecule metabolites in the physiological processes in organs, bloods, or tissue fluids of an organism. Metabolomics could comprehensively and holistically detect the metabolic changes to evaluate the efficacy of treatment, which makes it particularly suitable for the evaluation of the holistic and synergistic effects of herbal medicines.

In this study, the prevention effect of *C. nitidissima Chi* on colitis-associated carcinogenesis and the underlying mechanisms were investigated by a nuclear magnetic resonance (NMR)-based metabolomics approach. AOM/DSS-induced CRC, a classical CRC model was established, tissues of intestine, kidney, and spleen were collected for metabolomics analysis. Orthogonal-partial least squares-discriminant analysis (OPLS-DA) and correlation network analysis were applied to excavate differential metabolites and disturbed pathways between treatment groups. Pathological inspection suggested that *C. nitidissima Chi* could greatly relieve colonic pathology and neoplasm of CRC mouse model. NMR-based metabolomics analysis revealed that AOM/DSS induced severe metabolic disturbance which could be largely rectified by *C. nitidissima Chi*.

## Materials and methods

### Preparation of the extracts

The leaves of *C. nitidissima Chi* were provided by Guirentang Group Co., Ltd., Guangxi province, China and authenticated by plant taxonomist. The leaves were simmered in hot filtered water for 3 h. The clear solution was collected and evaporated using a rotary evaporator at 50°C to a volume of a half. The concentrated solution was extracted with water-saturated *n*-butanol, which was concentrated to dryness in vacuum and further lyophilized to obtain a yellow brown powder of *C. nitidissima Chi* extract (CNC group). The *C. nitidissima Chi* aqueous extract concentrated liquor (JHC group), a tea drink called Jin Hua Cha, were provided by Guirentang Group Co., Ltd., Guangxi province, China.

### Animals and experimental procedure

All animal experimental procedures were in accordance with the guidelines of the Instituted Animal Care and Use Committee of China Pharmaceutical University. ICR mice weighting 15–20 g (specific pathogen-free) were obtained from the jsj-lab laboratory animal co., LTD (license NO: SCXK(Hu)2012-0006). The animals were maintained under controlled conditions (22°C, 12 h/12 h dark/light cycle). There were 65 mice used in the study, 15 in normal control group, 25 in model group, 15 in JHC group, and 10 in CNC group.

**Figure 1:**
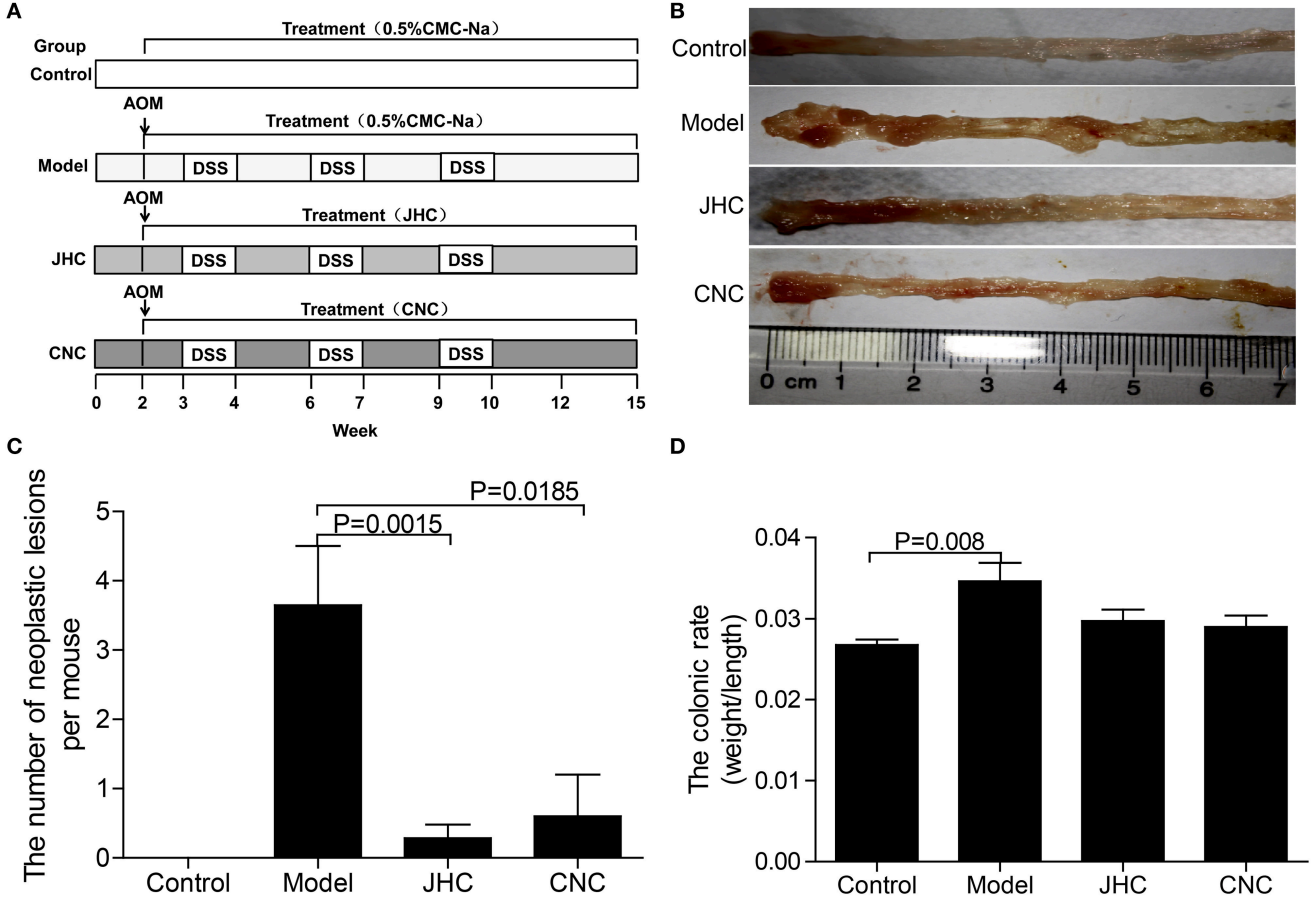
*Camellia nitidissima Chi* inhibited colitis-associated colon carcinogenesis induced by AOM/DSS. **(A)** Experimental protocol for colitis-associated colon carcinogenesis model. **(B)** Microscopic view of colon in mice after anatomy. **(C)** The number of neoplastic lesions in every group. **(D)** The colonic ratio (colon weight and colon length ratio) in every group after anatomy. Data are presented as mean ± SD vs. control and model.

Procedures of AOM/DSS induced CRC model was shown in Figure 1A. At week 2, mice were injected with AOM (10 mg/kg, i.p.). One week later, mice were firstly subjected to drinking water containing 2% DSS (MPBIO) for 1 week, and then followed by 2 weeks of purified water for recovery. The whole procedure was duplicated twice to establish the CRC model. The *C. nitidissima Chi* extract (CNC or JHC) (100 mg/kg) or equivalent 0.5% CMC-Na was administered by gavage using a soft tube once a day. After 10 weeks, some mice were sacrificed to observe the tumor genesis. At the 15th week, the mice survived in control, model, JHC, and CNC group were 15, 17, 14, and 10, respectively.

The mice were killed by cervical dislocation. In order to comprehensively reveal the metabolic response in different tissues under the AOM/DSS stress, and to evaluate the prevention effect of *C. nitidissima Chi* on AOM/DSS induced colorectal cancer, tissues of intestine, kidney, and spleen were collected. The colons were cut longitudinally along the main axis and macroscopically inspected the number of neoplastic lesions based on gross examination. Four mice were randomly selected from each group for pathological inspection to assess the prevention effect of CNC and JHC on colitis-associated carcinogenesis. Tissues of colon, kidney, and spleen were subjected to metabolomics analysis (*n* = 7, 16, 12, and 9 for intestines; *n* = 7, 14, 12, and 7 for kidneys; *n* = 10, 15, 11, and 9 for spleens in control, carcinoma, JHC, and CNC group, respectively). All the samples were flash-frozen in liquid nitrogen and stored at −80°C for further experiment.

### Histopathology and serum biochemistry

Fresh colons were immediately immersed in 10% paraformaldehyde for 24 h fixation, and then embedded in paraffin. Next, the tissues were stained with hematoxylin-eosin (HE) to examine the neoplastic lesions by a pathologist. The serum was collected by high speed centrifugation at 4°C. The activities of superoxide dismutase (SOD), catalase (CAT), and the contents of malondialdehyde (MDA) in serum were measured by assay kits from Nanjing Jiancheng Bioengineering Institute. The experiment was repeated three times.

### ^1^H NMR spectroscopy

Metabolites were extracted based on the reported protocols (Li et al., 2016). Briefly, tissues weighting about 200, 200, and 110 mg for intestine, kidney, and spleen were weighted, homogenized with an icy cold solvent (the ratio of tissue weight to 50% acetonitrile volume was 1: 5, mg/μL), vortexed, and centrifuged for 10 min at 12,000 g and 4°C. The supernatant was transferred into fresh Eppendorf tube and was then frozen and lyophilized to dryness on a vacuum concentrator. The dried samples were stored at −80°C until use. For NMR measurements, samples were dissolved in 550 μL 99.8% deuteroxide (D_2_O) phosphate buffer (0.2 M, pH = 7.4) containing 0.05% (w/v) sodium 3-(trimethylsilyl) propionate-2, 2, 3, 3-d4 (TSP). After vortexing and centrifugation, the supernatant was then transferred to a 5 mm NMR tube for ^1^H NMR analysis.

^1^H NMR spectra of samples were recorded on a Bruker AVANCE III 500 MHz NMR spectrometer at 298 K. D_2_O was used for field frequency locking and TSP was used as a chemical shift reference (^1^H, 0.00 ppm). A transverse relaxation-edited Carr-Purcell-Meiboom-Gill (CPMG) sequence [90(τ-180-τ) n-acquisition] with a total spin-echo delay (2 nτ) of 40 ms was used to suppress the signals of proteins. ^1^H NMR spectra were measured with 128 scans into 32 K data points over a spectral width of 10,000 Hz. The spectra were fourier transformed after multiplied the FIDs (free induction decay) by an exponential weighting function corresponding to a line-broadening of 0.5 Hz.

### Spectra pre-processing and multivariate data analysis

All ^1^H NMR spectra were phased, baseline-corrected, and aligned to TSP (0 ppm) manually in the Topspin software (version 2.1, Bruker), then were exported to ASCII files using MestReC (version 4.9.9.6, Mestrelab Research SL), which were then imported into R software (http://www.r-project.org) to do further phase and baseline correction and peak alignment. Each spectrum was binned using an adaptive, intelligent algorithm (De Meyer et al., 2008). The regions neighboring the residual water signal was removed. The total spectral area of the remaining bins was normalized a constant sum to facilitate the comparison between the spectra. Then, data transformation (generalized logarithm transformation) and Pareto scaling (mean-centered and divided by the square root of standard deviation of each variable) were used for univariate and multivariate analysis, respectively.

Principal component analysis (PCA) was first performed to detect any outliers and to show clusters between groups (Figure S1). From the PCA score plot of intestine, three apparent off-group points were found and were removed. To further investigate the therapeutic effects and excavate potential metabolic mechanism of CNC and JHC on treatment of colon cancer, OPLS-DA analysis was performed by using R software, which subtracted Y-uncorrelated X variations from the first PLS component, thus avoiding being circumvented by unwanted variation in the data set. The results were visualized by scores plots to show the classifications between groups, and corresponding loadings plots color-coded with absolute value of coefficients: the warm-colored (e.g., red) variables contributed more to intergroup differentiation than the cold-colored (e.g., blue) variables (**Figures 7, 8** and Figure S3). The validity of the models against over fitting was assessed by the parameters R^2^Y, and the predictive ability was described by Q^2^Y. To ensure that discrimination in the OPLS-DA model was not the result of data over-fitting, a validation of the model was further performed using permutation testing (2,000 times). The observed statistic *p* values *via* permutation testing which were less than 0.05 confirmed the significance of the OPLS-DA model at a 95% confidence level (Figure S2).

### Metabolites identification

Metabolites were assigned by querying some public metabolome databases such as Human Metabolome Database (Wishart et al., 2007), Madison-Qingdao Metabolomics Consortium Database (Cui et al., 2008), and commercial available software Chenomx NMR suite v.8.1 (Chenomx Inc., Edmonton, Canada), and finally confirmed by statistical total correlation spectroscopy techniques (STOCSY) (Cloarec et al., 2005) and by two-dimensional NMR techniques, e.g., total correlation spectroscopy (TOCSY) and heteronuclear single quantum correlation (HSQC).

### Univariate statistical analysis

A parametric Student's *t*-test or a non-parametric Mann-Whitney test (according to the conformity of the data to normal distribution) was performed on the integrated areas to evaluate the differences of metabolites between groups. The fold change (FC) values of metabolites and their associated *p*-values corrected by Benjamini-Hochberg adjusted method were calculated and visualized in colored tables. Fold change values were color encoded after log transformation. The cell unit was filled with red or blue colors to denote the increase or decrease of the metabolites in one group relative to the compared group.

### Shared and unique structures-plot analysis

When comparing two models, the interest of finding shared as well as unique compounds between two models is of great importance for the total understanding of the drug action. The use of the SUS-plot that combines information from a number of two-class models having the same reference is suggested as a solution (Wiklund et al., 2008). In the SUS-plot, the correlation from the predictive component, Corr(tp,X), of each model was plotted against each other. To improve clarity, all metabolites that were found not significant seen in the loading plot in either class were removed. The unique effects were found close to either the X or Y axis especially in colored frame, while the shared effects were located on the diagonals (**Figures 9A–C**). The Venn plot (**Figures 9D–F**) helped to visualize metabolites with positive and negative correlations between the two models as well as those belonged uniquely to specified class.

### Correlation network analysis

Pearson correlation networks of metabolites were calculated using the R package igraph (**Figures 10D–F, 11D–F**, 12D–F). In the networks, the nodes represent the metabolites, and the gray lines indicate that direct biological reactions exist between the connected nodes. Metabolites with correlation coefficients over 0.6 were joined with solid lines, colored in bluish to reddish corresponding to −1 to 1 of the correlation coefficients, whose widths were scaled according to their absolute values. SUS-plots were further used to filter out characteristic correlations between groups (**Figures 10A–C, 11A–C**, 12A–C).

## Results

### *Camellia nitidissima* chi inhibited colitis-associated carcinogenesis induced by AOM/DSS

AOM/DSS-induced CRC phenotypically resembled human CRC, thus was used in this study as CRC model. AOM/DSS induced a 100% colonic carcinogenesis rate in the model group, which was greatly reduced by the two treatment, only 14.29% in JHC and 10% in CNC group (Table 1, Figure 1B). Body weight loss of model mice were observed after 10 weeks compared with control group. CNC and JHC could apparently relieve the symptom at the end of the experiments. The number of neoplastic lesions decreased significantly after CNC and JHC treatment as compared with model group (Figure 1C). The colonic ratio (colon weight and colon length ratio) were significantly increased in the model group, which could be decreased by CNC or JHC treatment (Figure 1D). All the result suggested that CNC and JHC could inhibit colitis-associated colon carcinogenesis induced by AOM/DSS.

**Table 1:** *Camellia nitidissima Chi* inhibited the carcinogenesis lesions.

**Group**	**Mice sacrificed lesions**	**Mice with neoplastic lesions**	**Incidence of neoplastic lesions**	**Total neoplastic lesions**	**Average neoplastic lesions**
Control	15	0	0	0	0
Model	17	17	100%	57	3.35
CNC	10	1	10%	6	0.6
JHC	14	2	14.29%	4	0.29

### *Camellia nitidissima chi* relieved the colonic pathology and neoplasm of CRC mouse model

**Figure 2:**
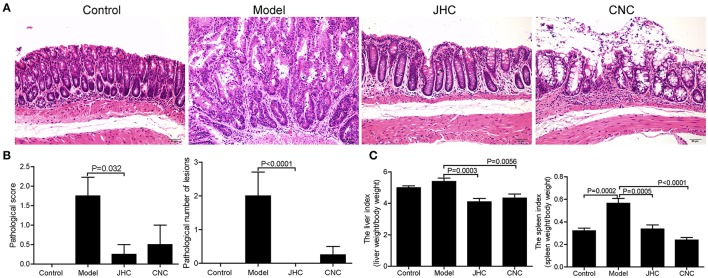
*Camellia nitidissima Chi* relieved the colonic pathology and neoplasm of CRC mouse model. **(A)** The colons were stained with HE (hematoxylin-eosin) to examine the neoplastic lesions. 200 × for all, scale bar = 50 μm. **(B)** The average pathological score and the pathological number of lesions in every group. Four slices were randomly selected from each group and stained with HE, then analyzed by the pathologist. **(C)** The liver index and spleen index in every group after anatomy.

The colons were stained with HE (hematoxylin-eosin) to examine neoplastic lesions (Figure 2A). Indicating tissue inflammation severity, the pathological scores (0–4, from normal to seriousness) (Geboes et al., 2000) were reduced significantly by CNC and JHC compared with model (Figure 2B). Moreover, the pathological number of lesions was decreased after two treatment, which is consistent with the visual gross examination result (Figure 2B). Finally, significant decreases of the liver index and spleen index also confirm the prevention effect of extracts (Figure 2C). These data demonstrated that CNC and JHC could relieve the colonic pathology of inflammation and neoplasm of CRC mouse model.

### *Camellia nitidissima chi* exhibited marked antioxidant effects

**Figure 3:**
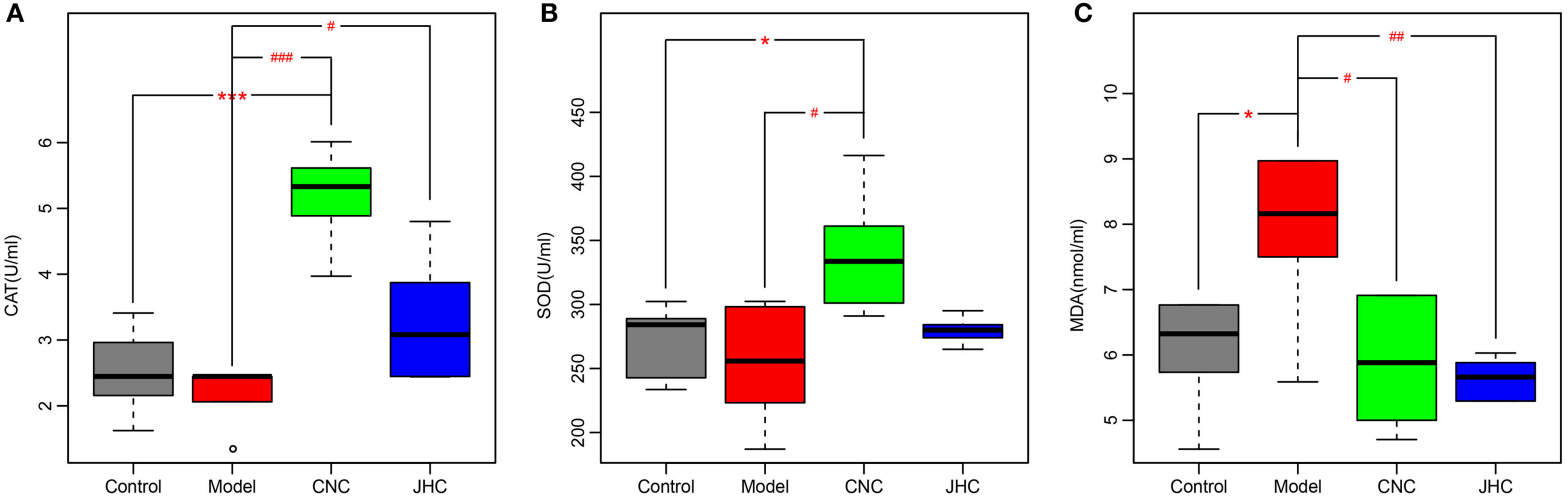
*Camellia nitidissima Chi* exhibited marked antioxidant effects. Boxplots for serum levels of CAT **(A)**, SOD **(B)**, and MDA **(C)**. The bottom and top of the box are the first and third quartiles, and the band inside the box is the second quartile (the median). The whiskers extend to ±1.5 times the interquartile range. ^*^p < 0.05 vs. control; ^***^p < 0.001 v*s*. control. ^#^*p* < 0.05 vs. model; ^*##*^*p* < 0.01 vs. model; ^*###*^*p* < 0.001 vs. model.

Catalase (CAT) and superoxide dismutase (SOD) are important antioxidant enzymes that protect organism from free radical induced damages. Their activities were significantly increased in treatment group compared with model, and even higher than control (Figures 3A,B). Moreover, CNC and JHC could markedly inhibit the product of lipid peroxide, MDA (Figure 3C). The serum biochemistry analysis suggested that CNC and JHC might prevent colitis-associated colon carcinogenesis via improving the body's antioxidant ability.

### Identification of metabolites and multivariate analysis

**Figure 4:**
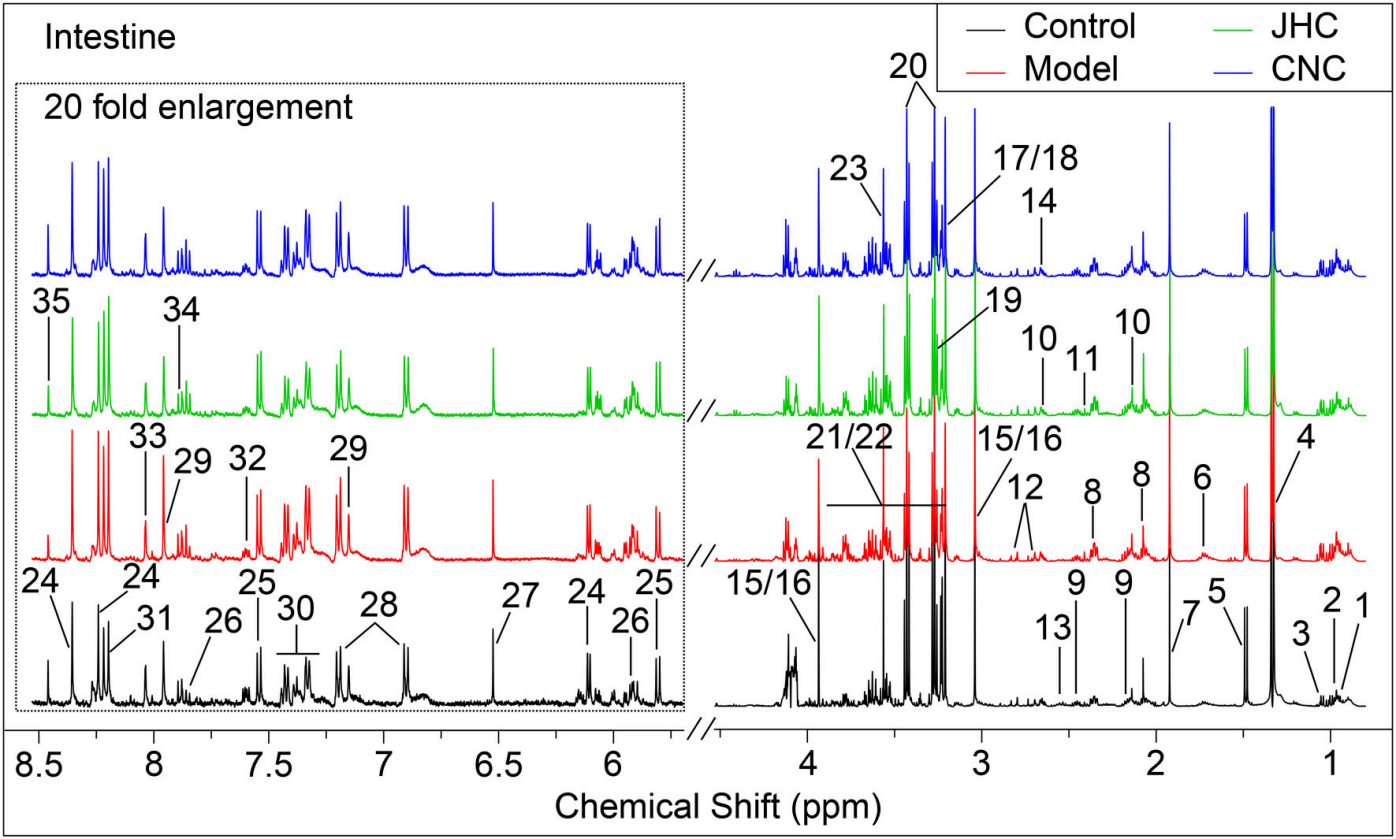
Typical 500 MHz ^1^H NMR spectra of intestine with the metabolites labeled. Metabolites: 1, Isoleucine; 2, Leucine; 3, Valine; 4, Lactate; 5, Alanine; 6, Lysine; 7, Acetate; 8, Glutamate; 9, Glutamine; 10, Methionine; 11, Succinate; 12, Aspartate; 13, Glutathione; 14, Dimethylamine; 15, Creatine; 16, PCr (phosphocreatine); 17, Choline; 18, OPC (O- phosphorylcholine); 19, Betaine; 20, Taurine; 21, Glucose; 22, Maltose; 23, Glycine; 24, Inosine; 25, Uracil; 26, Uridine; 27, Fumarate; 28, Tyrosine; 29, Histidine; 30, Phenylalanine; 31, Hypoxanthine; 32, Nicotinurate; 33, Guanosine; 34, UDP-galactose; 35, Formate.

**Figure 5:**
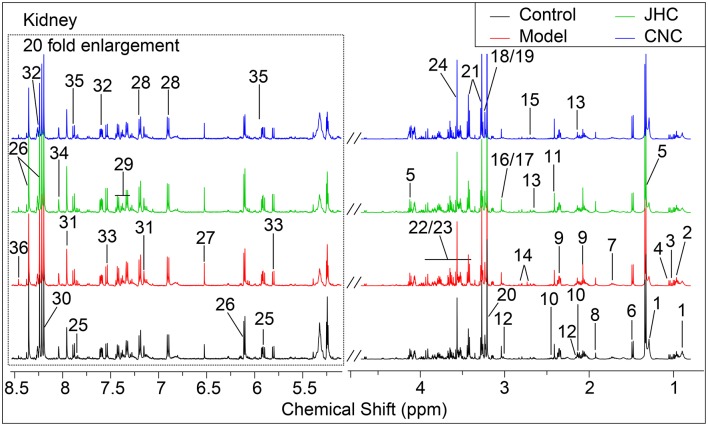
Typical 500 MHz ^1^H NMR spectra of kidney with the metabolites labeled. Metabolites: 1, Lipids; 2, Isoleucine; 3, Leucine; 4, Valine; 5, Lactate; 6, Alanine; 7, Lysine; 8, Acetate; 9, Glutamate; 10, Glutamine; 11, Succinate; 12, Glutathione; 13, Methionine; 14, Aspartate; 15, Dimethylamine; 16, Creatine; 17, PCr (phosphocreatine); 18, Choline; 19, OPC (O- phosphorylcholine); 20, Betaine; 21, Taurine; 22, Glucose; 23, Maltose; 24, Glycine; 25, Uridine; 26, Inosine; 27, Fumarate; 28, Tyrosine; 29, Phenylalanine; 30, Hypoxanthine;31, Histidine; 32, Nicotinurate; 33, Cytosine; 34, Guanosine; 35, UDP-galactose; 36, Formate.

**Figure 6:**
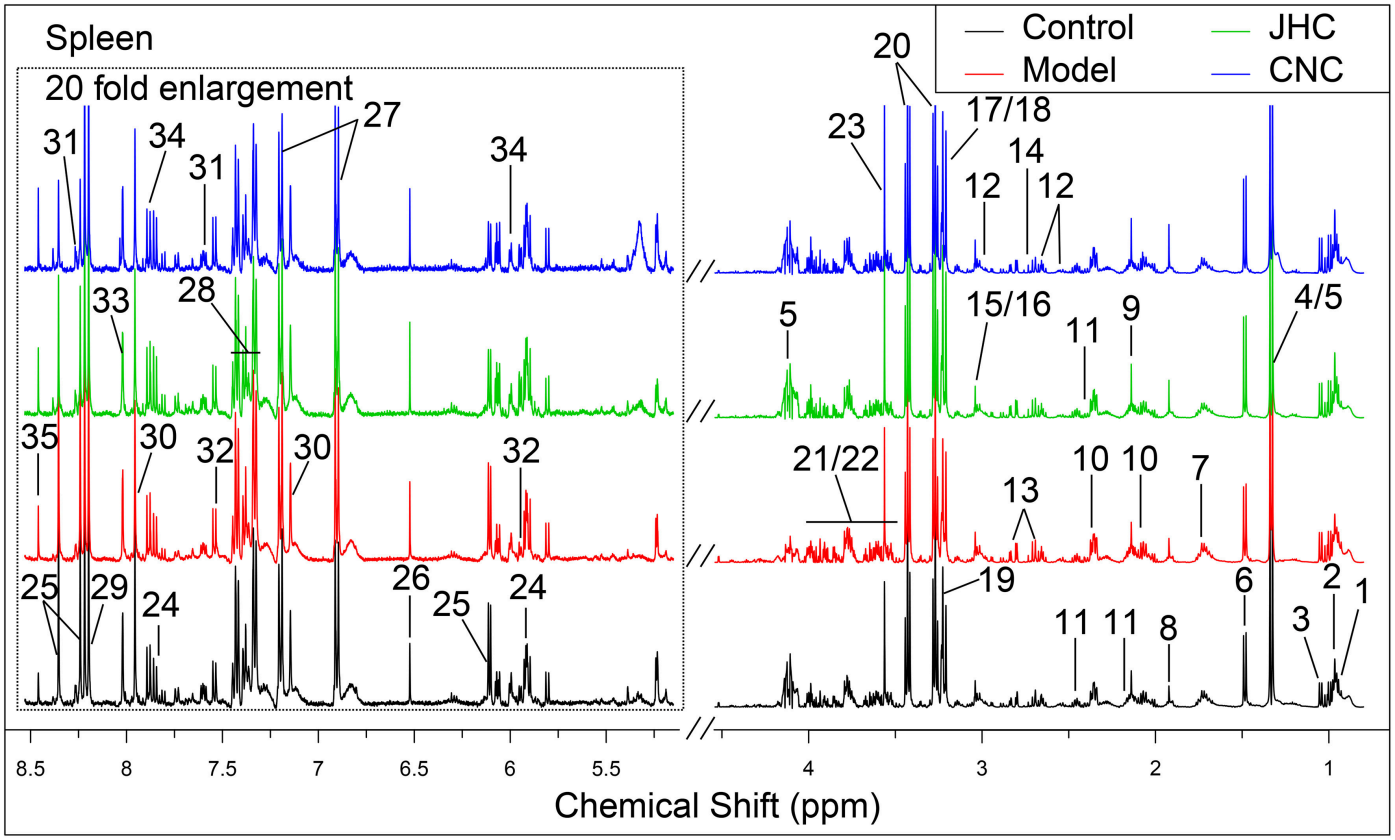
Typical 500 MHz ^1^H NMR spectra of spleen with the metabolites labeled. Metabolites: 1, Isoleucine; 2, Leucine; 3, Valine; 4, Lactate; 5, Threonine; 6, Alanine; 7, Lysine; 8, Acetate; 9, Methionine; 10, Glutamate; 11, Glutamine; 12, Glutathione; 13, Aspartate; 14, Dimethylamine; 15, Creatine; 16, PCr; 17, Choline; 18, OPC; 19, Betaine; 20, Taurine; 21, Glucose; 22, Maltose; 23, Glycine; 24, Uridine; 25, Inosine; 26, Fumarate; 27, Tyrosine; 28, Phenylalanine; 29, Hypoxanthine; 30, Histidine; 31, Nicotinurate; 32, Cytosine; 33, Guanosine; 34, UDP-galactose; 35, Formate.

Typical ^1^H NMR spectra for tissues of intestine, kidney and spleen were shown in Figures 4–6, respectively. A total of 35, 36, and 35 metabolites in the intestine, kidney, and spleen extracts were assigned, which is listed in Tables 2–4, respectively.

**Table 2:** Potential marker metabolites in mice intestine identified by ^1^H-NMR and their fold changes among groups and the associated *p*-values.

**No**.	**Metabolites**	**Model vs. Control**		**CNC vs. Model**		**JHC vs. Model**	
		**FC^a^**	***p*-value^b^**	**FC**	***p*-value**	**FC**	***p*-value**
1	Isoleucine	1.8	^***^	0.61	^***^	0.83	^*^
2	Leucine	1.92	^***^	0.58	^***^	0.73	^*^
3	Valine	1.58	^***^	0.63	^***^	0.87	
4	Lactate	0.84	^*^	1.23	^**^	0.93	
5	Alanine	1.07		1		0.92	
6	Lysine	1.63	^***^	0.7	^***^	0.75	
7	Acetate	1.8		0.7		0.75	
8	Glutamate	1.36	^*^	0.66	^**^	1.19	^***^
9	Glutamine	1.13		0.63	^***^	1.2	^*^
10	Methionine	1.67	^*^	1.2	^**^	0.97	^*^
11	Succinate	2.2	^**^	0.34	^***^	0.67	^*^
12	Aspartate	1.19		0.81		0.96	
13	Glutathione	0.52	^*^	1.38		1.53	
14	Dimethylamine	0.93		1.23		0.98	
15	Creatine	0.96		1.01		1.08	^*^
16	PCr	0.96		1.01		1.08	^*^
17	Choline	0.93		0.99		1.1	^*^
18	OPC	0.81	^*^	1.3	^***^	1.03	
19	Betaine	0.69	^*^	1.1	^*^	0.98	
20	Taurine	0.87		1.08		1.1	
21	Glucose	1.29	^***^	0.8	^***^	0.97	
22	Maltose	1.89	^**^	0.57	^***^	0.9	^*^
23	Glycine	1.3	^**^	1.1	^**^	1.09	
24	Inosine	1.05		0.92		0.85	
25	Uracil	1.05		1.02		0.99	
26	Uridine	1.2		0.82		0.92	
27	Fumarate	0.96		0.97		0.92	
28	Tyrosine	1.4		0.83		0.75	
29	Histidine	0.96		0.94		0.96	
30	Phenylalanine	1.17		0.83		0.71	
31	Hypoxanthine	1.42		1		1.05	
32	Nicotinurate	0.62	^*^	0.96		0.91	^*^
33	Guanosine	0.9		0.95		0.92	
34	UDP-galactose	0.95		0.98		0.89	
35	Formate	0.68		1.26		1.14	

a *Color coded according to the fold change value, red represents increased and blue represents decreased concentrations of metabolites*.

b *p-values corrected by BH (Benjamini Hochberg) methods were calculated based on a parametric Student's t-test or a nonparametric Mann-Whitney test (dependent on the conformity to normal distribution)*.

* *p < 0.05*,

** *p < 0.01*,

*** *p < 0.001*.

**Table 3:** Potential marker metabolites in mice kidney identified by ^1^H-NMR and their fold changes among groups and the associated *p*-values.

**No**.	**Metabolites**	**Model vs. Control**		**CNC vs. Model**		**JHC vs. Model**	
		**FC^a^**	***p*-value^b^**	**FC**	***p*-value**	**FC**	***p*-value**
1	Lipids	0.67	^**^	1.48		1.31	
2	Isoleucine	1.11		0.66	^***^	0.86	^***^
3	Leucine	1.23		0.74		0.88	^**^
4	Valine	0.95		0.42		1.06	
5	Lactate	0.87		0.94		1.02	
6	Alanine	1.15		0.93		0.97	
7	Lysine	1.2		0.7	^***^	0.91	
8	Acetate	1.18		0.82		1.28	^***^
9	Glutamate	0.95		0.82	^***^	1.1	
10	Glutamine	1.06		0.73	^***^	1.02	
11	Succinate	0.86		1.05		1.31	
12	Glutathione	1.05		0.93		1.02	
13	Methionine	1.15		0.72		0.91	
14	Aspartate	1.2		0.79	^***^	1.07	
15	Dimethylamine	3.03	^**^	0.48	^***^	0.32	^***^
16	Creatine	1.03		0.68	^***^	1.02	
17	PCr	1.03		0.68	^***^	1.02	
18	Choline	1.13		1.11	^***^	1.06	
19	OPC	0.93		0.92		1.03	
20	Betaine	1.04		1.09		0.95	
21	Taurine	0.98		1.09		1.08	
22	Glucose	0.71		0.77		0.95	
23	Maltose	1.25	^**^	0.72	^***^	0.97	
24	Glycine	1.18		0.98		1.02	
25	Uridine	1.17		0.88	^*^	1.17	
26	Inosine	0.99		0.69	^***^	1.14	
27	Fumarate	1.18		1.01		1.1	
28	Tyrosine	1.11		0.73		0.87	
29	Phenylalanine	1.21		0.85		0.93	
30	Hypoxanthine	1.14		0.75	^**^	1.11	
31	Histidine	1.15		0.85		1.17	
32	Nicotinurate	1.04		0.95		1.19	
33	Cytosine	1		0.9		1.29	
34	Guanosine	1.08		1.04		1.04	
35	UDP-galactose	0.98		0.96		1.15	
36	Formate	1.52		0.74			

a *Color coded according to the fold change value, red represents increased and blue represents decreased concentrations of metabolites*.

b *p-values corrected by BH (Benjamini Hochberg) methods were calculated based on a parametric Student's t-test or a nonparametric Mann-Whitney test (dependent on the conformity to normal distribution)*.

* *p < 0.05*,

** *p < 0.01*,

*** *p < 0.001*.

**Table 4:** Potential marker metabolites in mice spleen identified by ^1^H-NMR and their fold changes among groups and the associated *p*-values.

**No**.	**Metabolite**	**Model vs. Control**		**CNC vs. Model**		**JHC vs. Model**	
		**FC^a^**	***p*-value^b^**	**FC**	***p*-value**	**FC**	***p*-value**
1	Isoleucine	1.16		0.8	^***^	0.87	
2	Leucine	1.11		0.83	^*^	0.86	
3	Valine	1.09		0.91		0.89	
4	Lactate	0.81	^*^	1.3		1.21	^***^
5	Threonine	1.72	^*^	0.62	^***^	0.57	^***^
6	Alanine	0.99		0.94		1	
7	Lysine	1.07		0.96		0.92	
8	Acetate	1.04		1.34	^***^	1.03	
9	Methionine	1.09		0.89		0.92	
10	Glutamate	1.15	^***^	0.82	^***^	0.87	^***^
11	Glutamine	0.96		1.1	^*^	1.12	^**^
12	Glutathione	1.14		0.69	^**^	0.71	^***^
13	Aspartate	1.08		0.78	^**^	0.89	^*^
14	Dimethylamine	1.04		0.96		1.02	
15	Creatine	1.12		0.88		0.81	^***^
16	PCr	1.12		0.88	^*^	0.81	^***^
17	Choline	0.95		1.14	^*^	1.16	^***^
18	OPC	1.07		0.96		1.05	
19	Betaine	0.88		1.03		1.14	^*^
20	Taurine	0.94		1.07		1.08	^**^
21	Glucose	1.24	^**^	0.8	^*^	0.8	^***^
22	Maltose	1.24	^**^	0.8	^*^	0.8	^***^
23	Glycine	0.91		1.12		1.17	^*^
24	Uridine	0.96		1.05		1.14	
25	Inosine	0.87		0.67	^***^	0.82	^***^
26	Fumarate	0.94		1.05		1.04	
27	Tyrosine	0.97		0.93		0.98	
28	Phenylalanine	0.96		0.88		0.93	
29	Hypoxanthine	1.03		0.92		0.96	
30	Histidine	1.07		1.03		0.98	
31	Nicotinurate	0.71	^*^	1.25		1.23	
32	Cytosine	0.85		1.19		1.05	
33	Guanosine	0.83		1.5		2.22	
34	UDP-galactose	0.89		0.9		1.1	
35	Formate	1.02		1.53		1.18	

a *Color coded according to the fold change value, red represents increased and blue represents decreased concentrations of metabolites*.

b *p-values corrected by BH (Benjamini Hochberg) methods were calculated based on a parametric Student's t-test or a nonparametric Mann-Whitney test (dependent on the conformity to normal distribution)*.

* *p < 0.05*,

** *p < 0.01*,

*** *p < 0.001*.

**Figure 7:**
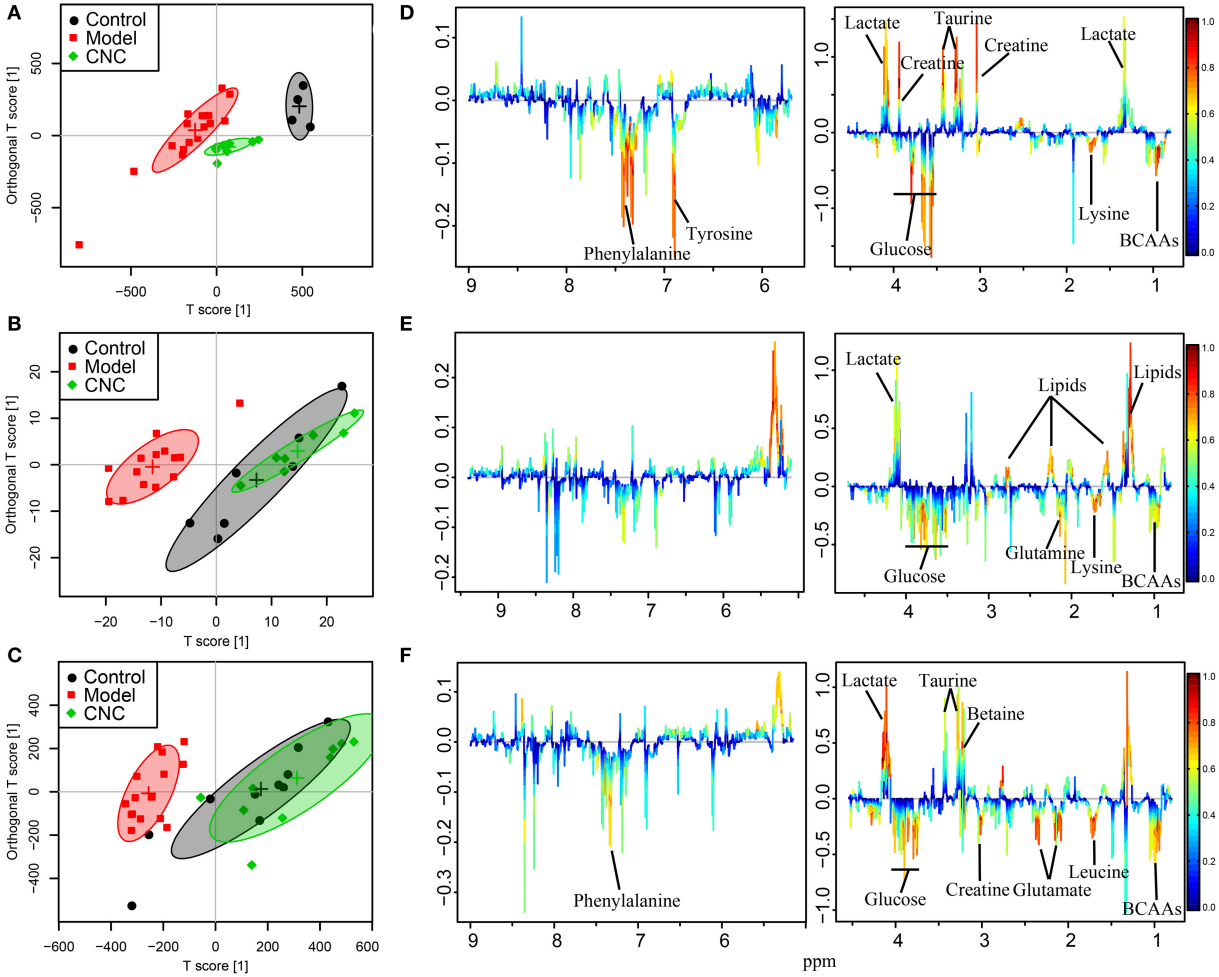
OPLS-DA analyses of metabolic profiles between control, model and the CNC groups for intestine, kidney, and spleen. Score plots **(A)** and color-coded coefficient loadings plots **(D)** for intestine (*R*^2^ = 0.96, *Q*^2^ = 0.90). Score plots **(B)** and color-coded coefficient loadings plots **(E)** for kidney (*R*^2^ = 0.90, *Q*^2^ = 0.68). Score plots **(C)** and color-coded coefficient loadings plots **(F)** for spleen (*R*^2^ = 0.75, *Q*^2^ = 0.62). Significantly changed metabolites were assigned in the loadings plots. Negative signals represent increased and positive signals represent decreased concentrations in model group. Symbols of • (black filled circles), 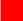 (red filled squares), and 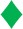 (green filled rhombuses) represent the control, model, and CNC groups, respectively.

**Figure 8:**
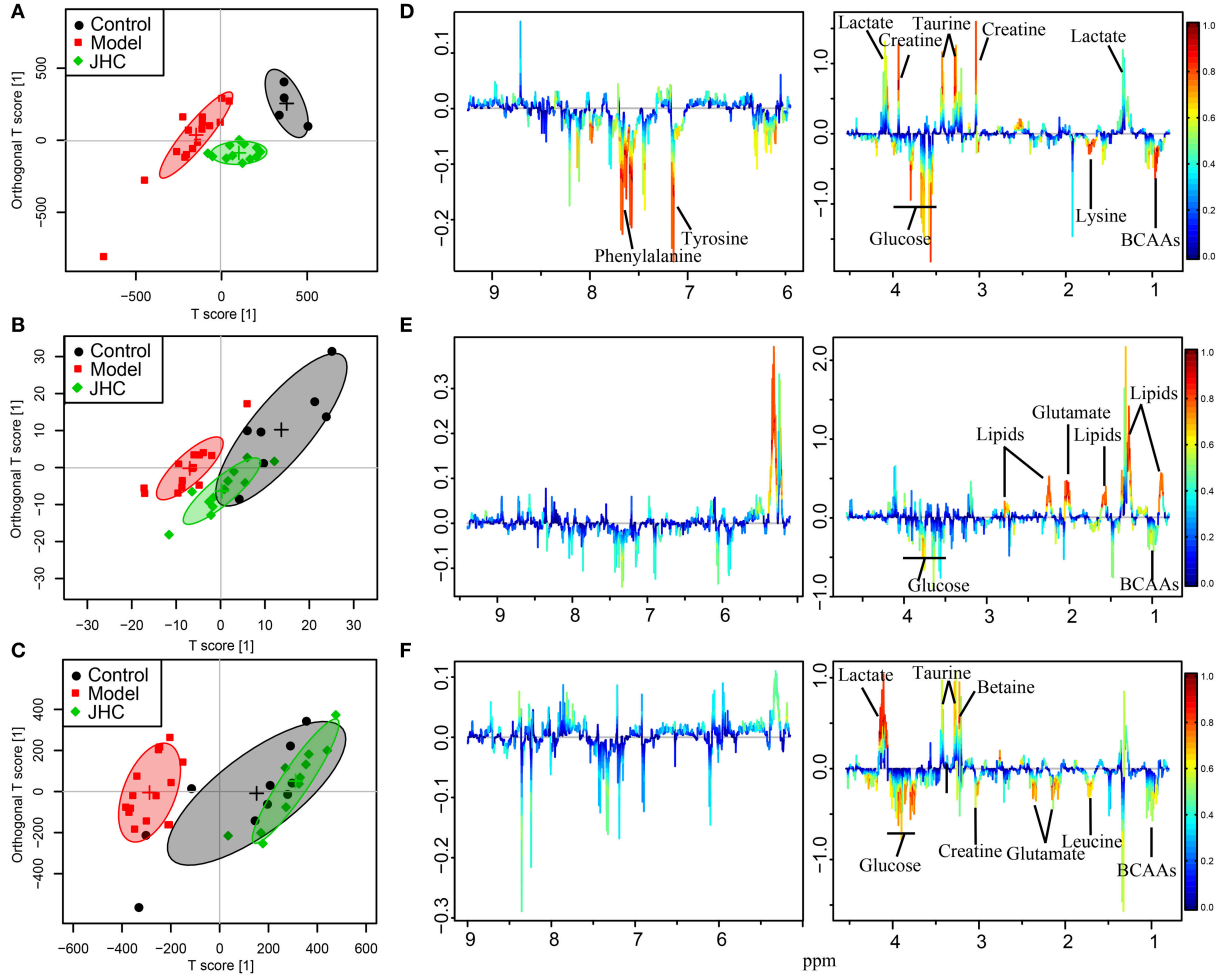
OPLS-DA analyses of metabolic profiles between control, model and the JHC groups for intestine, kidney, and spleen. Score plots **(A)** and color-coded coefficient loadings plots **(D)** for intestine (*R*^2^ = 0.80, *Q*^2^ = 0.63). Score plots **(B)** and color-coded coefficient loadings plots **(E)** for kidney (*R*^2^ = 0.65, *Q*^2^ = 0.49). Score plots **(C)** and color-coded coefficient loadings plots **(F)** for spleen (*R*^2^ = 0.82, *Q*^2^ = 0.56). Significantly changed metabolites were assigned in the loadings plots. Negative signals represent increased and positive signals represent decreased concentrations in model group. Symbols of • (black filled circles), 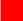 (red filled squares), and 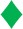 (green filled rhombuses) represent the control, model, and JHC groups, respectively.

In the OPLS-DA scores plots, model group and normal control group were clearly separated for intestine (Figures 7A, 8A), kidney (Figures 7B, 8B), and spleen (Figures 7C, 8C), indicating that AOM/DSS treatment severely disturbed the metabolic profiling of both targeted and non-targeted organs. The pretreated groups were severely overlapped with the control group in the OPLS-DA scores plots of spleen and kidney, while the pretreated groups were in between of model and control groups in the OPLS-DA scores plots of intestine, showing the superior comprehensive protection of *C. nitissima Chi* against poisoning of AOM/DSS in multi-organs. The results suggested a significant remedial effect of *C. nitissima* Chi on related tissues concerning colon cancer, indicating its superior health beneficial function to organism. Hence, the multi-tissue aspect of this study is clearly a strength, which could gain the knowledge for us a more comprehensive understanding of the mechanism of the action of *C. nitissima Chi* in prevention of the AOM/DSS induced colorectal cancer.

The loadings plots were color-encoded according to the correlation coefficients (r^2^) and visualized in a covariance-based pseudo-spectrum. The weight of a variable in the discrimination model was given by the square of its r^2^, which was color coded from zero in blue to one in red. The color-coded loadings plots represented metabolites for intestines (Figures 7D, 8D), kidneys (Figures 7E, 8E), and spleens (Figures 7F, 8F), respectively.

### SUS-plot analysis

**Figure 9:**
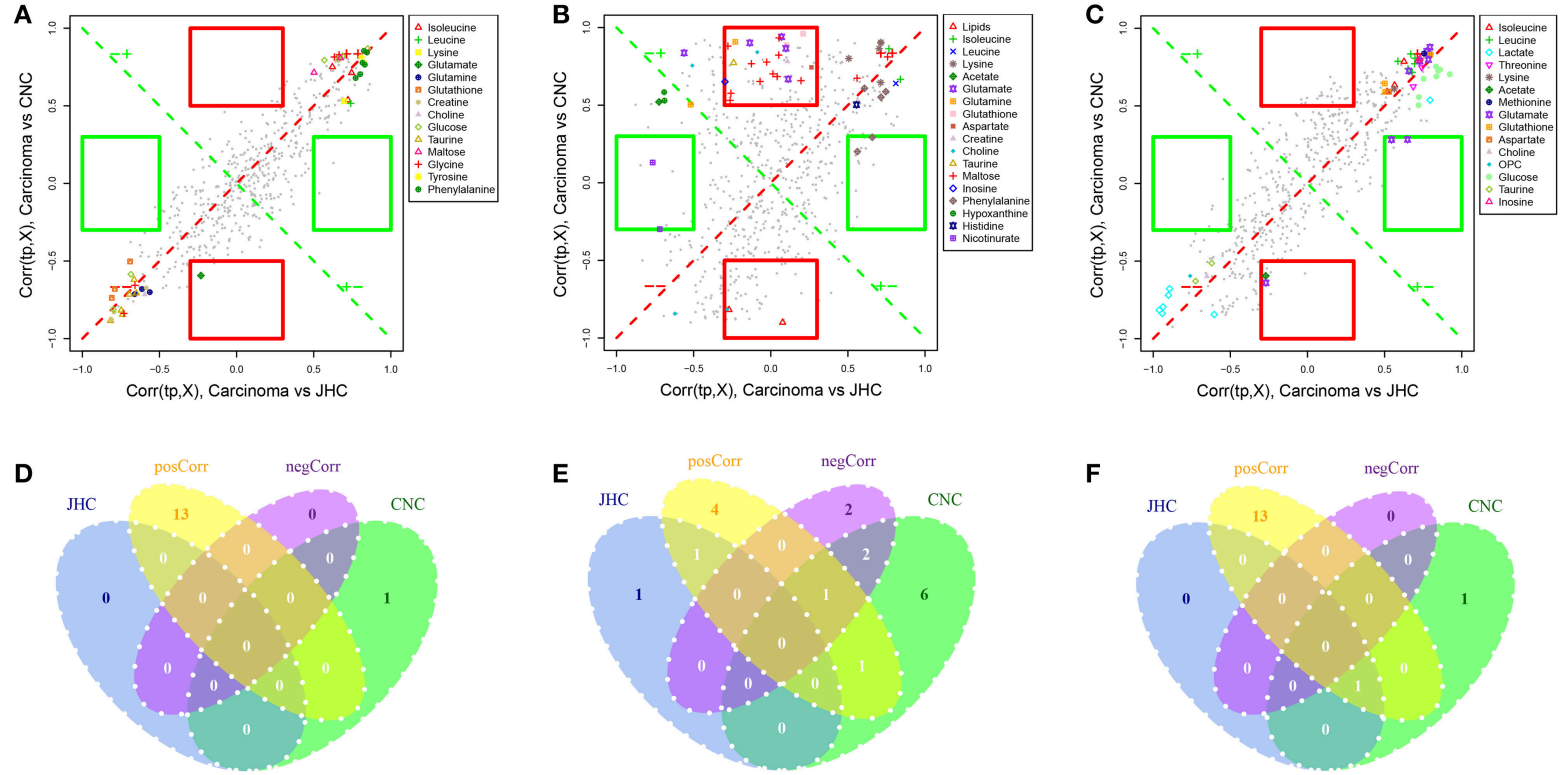
Shared and unique structures (SUS)-plot analysis correlating the OPLS-DA models of carcinoma vs. JHC and carcinoma vs. CNC. The metabolites close to the red diagonal line **(A–C)** are equally affected in both groups, and the metabolites in the rectangular frame were uniquely affected in a single group. The Venn plot **(D–F)** was used to illustrate the metabolites that were shared between classes and their positive and negative correlations as well as those that belong to only a single class.

In the SUS-plots (Figures 9A–C) and the Venn plots (Figures 9D–F), 13 metabolites close to the red diagonal line (positive correlation) were shared metabolites of the two models for intestine, such as isoleucine, leucine, lysine, and *etc*., indicating that CNC and JHC shared the common targets and metabolic pathways related with their antineoplastic activity. The metabolite in red frame of kidney tissues (Figure 9B) were unique in CNC group, such as lipids, choline, glutathione, taurine, glutamine, glutamate, maltose, aspartate, and creatine. Lipids were significantly decreased in kidney of the model group. AOM/DSS treatment could disturbed the balance of oxidation reduction equilibrium, causing the formation of massive free radical, or ROS. Cell membrane is particularly susceptible to continuous accumulation of free radicals for its abundance in lipids, which were prone to be attacked by ROS. As the precursors of all membrane phospholipids, choline can be utilized for the construction of damaged membranes. Glutathione and taurine are important anti-oxidation metabolites in the body. The increased levels of glutathione and taurine indicated that CNC and JHC exhibited marked antioxidant effects, which was in accordance with the serum biochemical assays. The SUS-plot of kidney revealed that CNC could more extensively and efficiently improve the anti-oxidation status of kidney than JHC to attenuate the oxidative damages caused by AOM/DSS.

## Discussions

Pharmacodynamics evaluation combined with multivariate statistical analysis and correlation network analysis revealed that AOM/DSS-induced CRC showed marked changes of metabolites concerning glycolysis, amino acids metabolisms, glutamine, and glutamate metabolism, oxidative stress as well as nucleic acid metabolism, which could be reversed toward the normal state by *C. nitidissima Chi*.

### Glycolysis

Glucose is catabolized *via* glycolysis to pyruvate, producing adenosine triphosphate (ATP). Pyruvate could be further transported into mitochondria to produce ATP through TCA cycle and the respiratory electron-transport chain, which is more efficient, generating more ATP than glycolysis. Normal cells produce ATP primarily through TCA cycle by mitochondrial oxidative phosphorylation; while cancer cells produce ATP via aerobic glycolysis, known as the Warburg effect (Koppenol et al., 2011). The level of lactate in intestine of model group was significantly decreased, while the level of succinate was markedly increased. Lactate is the end-product of glycolysis, therefore, its decease suggested an inhibited glycolysis; while the increase of succinate, an intermediate of the TCA cycle, indicated an enhanced TCA cycle in colorectal carcinoma to afford the energy requirement of tumor proliferation. Glucose was significantly increased in intestines of model group, which was also favorable for the survival of the tumor cells in energy deficit. Taken together, oxidative phosphorylation may play more important role in energy production in CRC cancers than glycolysis in most tumors (Zu and Guppy, 2004).

However, the level of lactate in intestine of *C. nitidissima Chi* pretreated group was significantly elevated, and the level of glucose was significantly decreased. The reason maybe that CNC could significantly inhibit the uptake and utilization of glucose and block the TCA cycle and oxidative phosphorylation in tumor cells. The tumor cells had to utilize inefficient glycolysis for energy supply. Pretreatment of *C. nitidissima Chi* produced a net effect of insufficient energy supply in CRC cells to meet the huge energy demand during hyperplasia, thus inhibiting the growth and proliferation of tumor cells, exhibiting anti-tumor effects.

### Amino acids metabolisms

**Figure 10:**
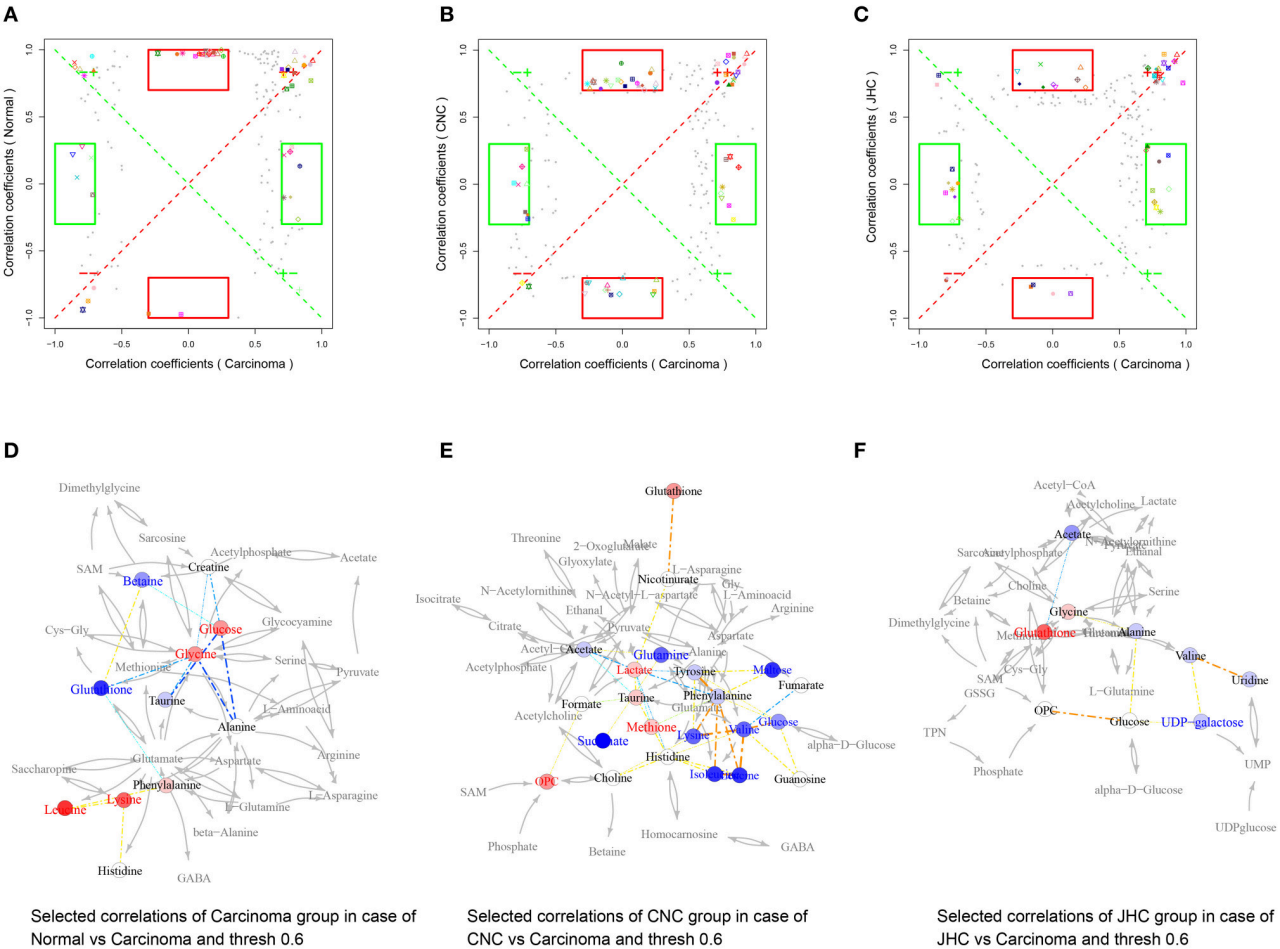
Metabolic correlation network analysis for intestine tissues. SUS-plots were used to filter out characteristic correlations between groups **(A–C)**. Metabolites with coefficients of Pearson's correlations that were above a threshold are connected by solid lines that are color-coded according to the values of the coefficients (a warm color represents a positive correlation, and a cool color represents a negative correlation). The width of each line is scaled based on its absolute values. The names of the metabolites shown in red and blue indicate that they were significantly increased and decreased in model **(D)**, CNC **(E)**, and JHC **(F)** groups, respectively. The gray lines between the metabolites indicate direct biological reactions.

Obvious correlations among BCAAs (leucine, isoleucine, and valine) were exhibited in Figure 10E, indicating severe metabolic changes of them. The levels of branched chain amino acids (BCAAs, leucine, isoleucine, and valine) were remarkably elevated in the model group in intestines, which is in accordance with an early event in human pancreatic adenocarcinoma development (Harper et al., 1984). BCAAs are essential amino acids that have to be obtained by dietary intake or protein degradation. A number of studies have demonstrated that marked alterations in BCAA metabolism were found in cancer (Baracos and Mackenzie, 2006). Rapid, dysregulated cell growth in cancer cells calls for extra sources of energy and nutrients like glucose and essential amino acids. The increase of BCAAs could firstly meet the demands of *de novo* protein synthesis in the proliferation of tumorous tissue. BCAAs can also be utilized for energy production through a catabolic pathway mediated by branched-chain amino acid aminotransferase (BCAT). BCAAs provides acetyl-CoA and/or anaplerotic substrates for TCA cycle (Harper et al., 1984; Tokunaga et al., 2004). Population-based human studies have demonstrated that elevated plasma BCAAs correlate with pancreatic cancer risk (Tönjes et al., 2013; Mayers et al., 2014), which reflected the role of BCAAs as a fuel reservoir. In addition, increasing evidence shows that BCAAs, especially leucine, are also nutrient signals regulating the mammalian target of rapamycin (mTOR) pathway (Avruch et al., 2009; Nicklin et al., 2009), which is recognized as a critical regulator of cellular function (Laplante and Sabatini, 2009; Waickman and Powell, 2012) such as protein transcription, and cell growth, proliferation, and autophagy. Chiharu Tokunaga proposed that leucine modulates mTOR function, in part by regulating mitochondrial function and AMP-activated protein kinase (Tokunaga et al., 2004). The pathway of mTOR is upregulated in many types of cancerand mTOR-targeted cancer therapy has been successfully applied in clinic (Advani, 2010). Previous study (Tönjes et al., 2013) demonstrated that BCAT and BCAA metabolism are attractive targets for the development of therapeutic approaches to treat glioma patients. Altogether, given the rapid growth of cancer and the metabolic reprogramming that occurs during the tumor development (Hanahan and Weinberg, 2000, 2011), elevated levels of BCAAs could be utilized for de novo protein synthesis as well as be used as a source of energy during proliferation of tumorous tissue. Pretreatment of *C. nitissima* Chi significantly decreased levels of BCAA in intestine, kidney, and spleen tissues, which should contribute to its tumor preventive effect.

A positive correlations between methione and taurine was observed in Figure 10E. Methionine is a dietary indispensable amino acid required for normal growth and development, serving as the major methyl group donor *in vivo*. Previous research indicated that people consuming more methionine were at a reduced risk of developing colorectal cancer (Chen et al., 1996), and inadequate intakes of methionine may increase risk of colon cancer (Giovannucci et al., 1995). Moreover, liver cancer could be induced by dietary deficiency of choline and methionine without added carcinogens (Ghoshal and Farber, 1984). Thus, clearly, the correlation network of intestine offers an intriguing new clue for elucidating the ameliorative effects of CNC and JHC on CRC process.

**Figure 11:**
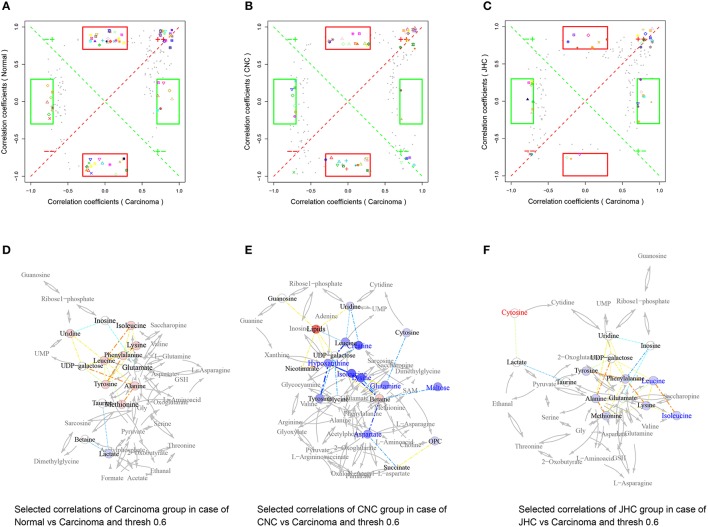
Metabolic correlation network analysis for kidney tissues. SUS-plots were used to filter out characteristic correlations between groups **(A–C)**. Metabolites with coefficients of Pearson's correlations that were above a threshold are connected by solid lines that are color-coded according to the values of the coefficients (a warm color represents a positive correlation, and a cool color represents a negative correlation). The width of each line is scaled based on its absolute values. The names of the metabolites shown in red and blue indicate that they were significantly increased and decreased in model **(D)**, CNC **(E)**, and JHC **(F)** groups, respectively. The gray lines between the metabolites indicate direct biological reactions.

In the correlation network of kidney between JHC and carcinoma group (Figure 11F), phenylalanine located in the center, showing positive correlations with many other amino acids such as tyrosine, leucine, lysine, and methionine. Pervious study demonstrated that decreased phenylalanine-tyrosine intake could suppress tumor growth in patients with advanced malignant melanoma. Possible reasons might be that deprival of these vital amino acids inhibited the protein synthesis and growth of tumors (Demopoulos, 1966).

**Figure 12:**
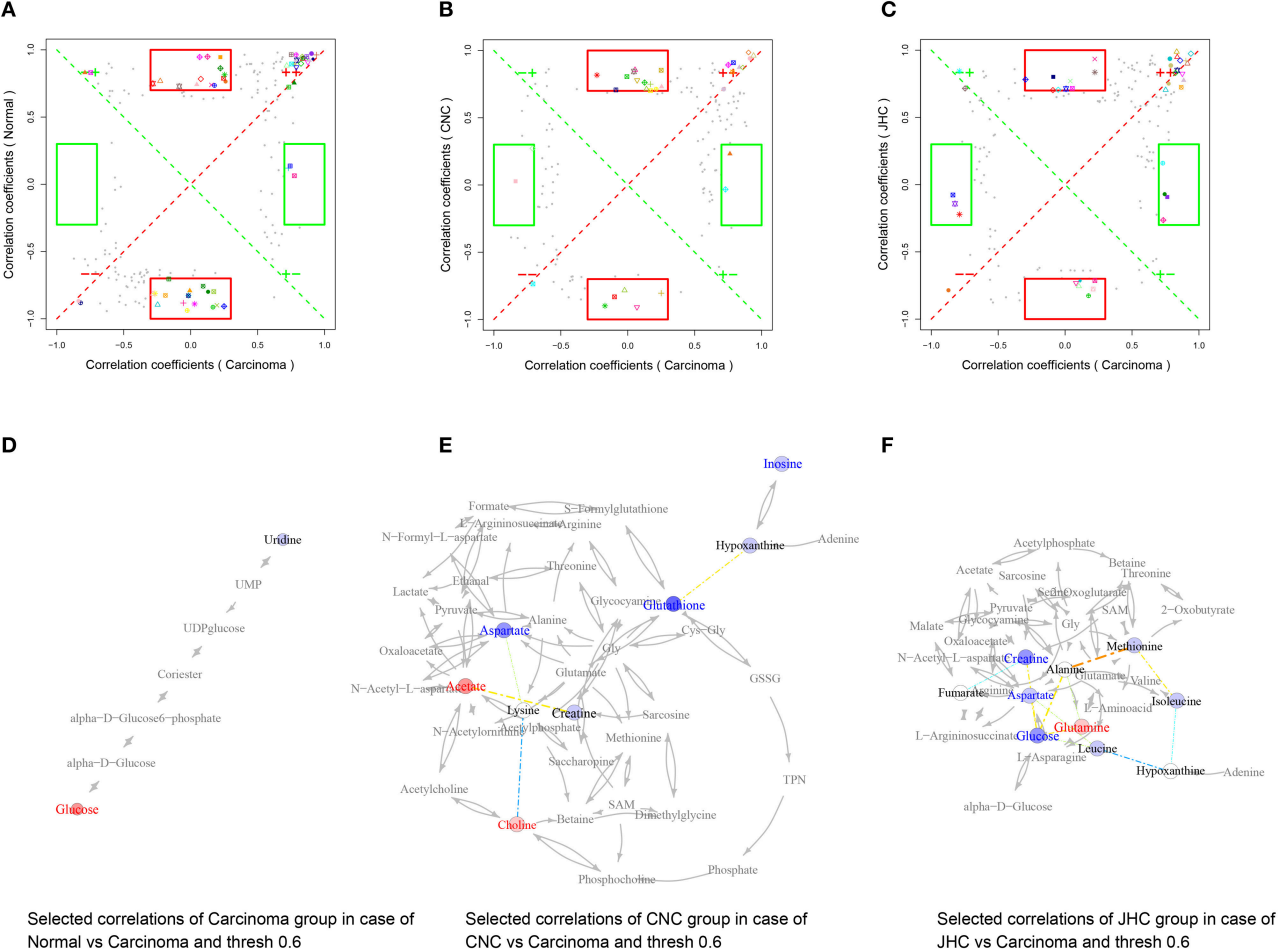
Metabolic correlation network analysis for spleen tissues. SUS-plots were used to filter out characteristic correlations between groups **(A–C)**. Metabolites with coefficients of Pearson's correlations that were above a threshold are connected by solid lines that are color-coded according to the values of the coefficients (a warm color represents a positive correlation, and a cool color represents a negative correlation). The width of each line is scaled based on its absolute values. The names of the metabolites shown in red and blue indicate that they were significantly increased and decreased in model **(D)**, CNC **(E)**, and JHC **(F)** groups, respectively. The gray lines between the metabolites indicate direct biological reactions.

### Glutamine and glutamate metabolism

The levels of glutamine and glutamate in intestine were significantly elevated in CRC model group. Cancer cells have a greatly increased demand for glutamine as preferred nitrogen donor (Mider, 1951; Durán et al., 2012), which are essential for cell proliferation (Wise and Thompson, 2010). The alteration of glutamine is one of the altered metabolic change that occurs in central metabolism of many cancer cells (Yoshikawa et al., 2006; Lu et al., 2010; Lunt and Vander Heiden, 2011). Glutamine can be metabolized via glutaminase to glutamate and further converted to the TCA cycle intermediate α-ketoglutarate, which provides an important entry point of carbon to fuel the TCA cycle (Jones and Thompson, 2009), thus contributing to the survival of proliferating cells (Wise and Thompson, 2010). The conversion of glutamine to glutamate was catalyzed by glutaminase, which was overexpressed in both solid tumors and many tumor cell lines. Tumor suppressor (p53) exerted its function by increasing the expression of mitochondrial isoform of glutaminase-2 (Hu et al., 2010). Therefore, inhibiting glutamine metabolism has been proposed as an anticancer therapy (Wise and Thompson, 2010).

Glutamate is a major bio-energetic substrate for proliferation of normal and neoplastic cells (Matés et al., 2002). Glutamate stimulated the proliferation of carcinoma cells in serum-deprived medium or medium supplemented with serum-replacement medium. Deprival of glutamate inhibited division and migration of tumor cells, enhanced cell death and altered morphology of tumor cells *in vitro*, resembling those produced by cytostatic drugs (Rzeski et al., 2001).

Significant increases of glutamine and glutamate in intestine of CRC model group revealed that AMO/DSS treatment create an environment suitable for tumor proliferation. CNC treatment could significantly decreased levels of glutamine and glutamate in intestine, which might contribute to the anti-tumor effects of *C. nitissima chi*.

### Oxidative stress

Oxidative stress played an important role in carcinogenesis (Leufkens et al., 2012). Increased oxidative stress is a common feature observed in many different types of tumors (Storz, 2013). It has been suggested that ROS participate in tumor progression by promoting DNA damage and/or altering cellular signaling pathways (Kang et al., 2012). When free radicals are produced in excessive and uncontrollable amounts, they and their derivatives may react with various cellular macromolecules, such as lipids, proteins, and DNA. A growing number of evidences revealed that ROS was implicated in a range of diseases, and might be important progenitors in CRC carcinogenesis (Perše, 2013). A cohort-nested case-control study on cancer and nutrition performed in Europe found that some biomarkers of oxidative stress could denote the risk of developing colorectal cancer (Leufkens et al., 2012). The antioxidant defense system is known to be composed of numerous antioxidants including primary (SOD, catalase, glutathione peroxidase, glutathione reductase), secondary (vitamin E, vitamin C, beta-carotene, uric acid, bilirubin, and albumin), and tertiary (biomolecules damaged by free radicals) ones.

Correlation among glutathione, taurine, and betaine in the carcinoma group were observed (Figure 10D). AOM/DSS administration could cause colorectal cancer by inducing severe oxidative stress and triggering radical inflammation reactions. The decreased levels of taurine, betaine, and glutathione in intestine of carcinoma group could be attributed to their depletion which were used by organism to fight against the great oxidative stress. Previous researchers reported that *C. nitidissima Chi* exhibited stronger antioxidant and anti-inflammation activity than other Theaceae plants and even tea polyphenols (Song et al., 2011). Moreover, we found that *C. nitidissima Chi* significantly elevated the activities of antioxidase, SOD, and CAT, and markedly decreased the level of MDA, suggesting that *C. nitidissima Chi* could prevent the colitis-associated colon carcinogenesis *via* improving the body's antioxidant ability. In addition, negative correlation between glycine and glutathione was found. Glycine could be transferred to glutathione under the catalysis of glutathione synthase, hence, the increased glycine and decreased glutathione could also be caused by the blocking of glutathione synthase by AOM/DSS, causing the accumulation of glycine and the deficiency of glutathione, causing excess ROS. Previous study also revealed that glutathione could control increased levels of ROS driven by increased cancer cell proliferation (Cairns et al., 2011), providing a strong evidence to our conclusion.

### Nucleic acid metabolism

Negative correlations between hypoxanthine and amino acids were found (Figure 11E), with hypoxanthine located in the pivot of the network, showing a central role of hypoxanthine. High concentrations of hypoxanthine were observed in patients with locally advanced rectal cancer (Kim et al., 2015). Hypoxanthine is known to be associated with various cancers, elevated hypoxanthine has been reported in the plasma of patients with acute lymphoblastic leukemia or non-Hodgkin's lymphoma (Hashimoto et al., 1992). The underlying mechanism for the change in hypoxanthine may be alterations of purine metabolism that occur during tumor development. A recent study has shown that urinary hypoxanthine is significantly increased when tumor development in mesothelioma-transplanted nude mice was maximized (Buhl et al., 1985). In addition, changes in the activity or expression of enzymes involved in hypoxanthine or xanthine metabolism, which might affect hypoxanthine and xanthine levels in NHL urines, cannot be ruled out. For example, xanthine oxidoreductase, a key enzyme in the degradation of DNA and RNA, is associated with histological grade of differentiation and severity of disease in colorectal cancer (Linder et al., 2009), as well as the migratory activity of human breast cancer cells (Linder et al., 2005; Fini et al., 2008). The level of hypoxanthine in intestine and kidney of CRC group was elevated, which was decreased after CNC treatment, showcasing the therapeutic potential of CNC on colorectal cancer.

### Efficacy comparison of CNC and JHC

As can be seen from Figure 3, levels of CAT and SOD were much higher in CNC group than in JHC group, indicating more potent ability of CNC to improve the anti-oxidative activity of the mice than JHC. According to the color Table 2, the SUS-plot and the correlation network, it could be concluded that CNC could more effectively reverse the abnormal metabolic status toward a normal condition. Therefore, anti-tumor components of *C. nitissima Chi* should not be those highly hydrophilic compounds, such as macro-molecules, saccharides, amino acids, polypeptides in high polar fraction. Effective components of *C. nitissima Chi* should be compounds of high polarity and butanol extractable. CNC has better effects than JHC because effective components after butanol extraction were enriched in CNC. Further in-depth investigation on the anti-tumor components of *C. nitissima Chi* would be warranted.

In summary, metabolomics analysis combined with correlation network analysis revealed that AOM/DSS-induced CRC showed marked changes of metabolites concerning glycolysis, amino acids metabolisms, glutamine, and glutamate metabolism, oxidative stress as well as nucleic acid metabolism, which could be reversed toward the normal state by *C. nitidissima Chi*. CNC exhibited a more potent antioxidant and preventive effect than JHC. To our best knowledge, this is the first report that *C. nitidissima Chi* could inhibit colitis-associated colon carcinogenesis and the pioneer study using metabolomics to evaluate the efficacy of *C. nitidissima Chi*. Metabolomics demonstrated its ability and feasibility to explore the pharmacodynamics of herbal medicine, and to uncover the intricate mechanisms of the interactions between medicines and organisms.

## Ethics statement

This study was carried out in accordance with the recommendations of the Instituted Animal Care and Use Committee of China Pharmaceutical University. The protocol was approved by the the Instituted Animal Care and Use Committee of China Pharmaceutical University.

## Author contributions

Conceived and designed the experiments: ML, HD, SY, LS, and JW. Performed the experiments: ML, HD, GK, LL, XL, SL, and AJ. Analyzed and interpreted the data: ML, HD, GK, LL, XL, SL, AJ, and JW. Drafted and revised the work: ML, HD, GK, LL, XL, SL, AJ, SY, LS, and JW. Final approval of the version to be published: ML, HD, GK, LL, XL, SL, AJ, SY, LS, and JW. Agreement to be accountable for all aspects of the work: ML, HD, GK, LL, XL, SL, AJ, SY, LS, and JW.

### Conflict of interest statement

The authors declare that the research was conducted in the absence of any commercial or financial relationships that could be construed as a potential conflict of interest.
