# Supplementary material for: Nuclear Magnetic Resonance-Based Metabolomics Approach to Evaluate the Prevention Effect of Camellia nitidissima Chi on Colitis-Associated Carcinogenesis
Source: Front Pharmacol. 2017 Jul 11;8:447. doi: 10.3389/fphar.2017.00447 (PMC5504182; doi:10.3389/fphar.2017.00447)
Supplement: Supplementary file 1 [file DataSheet1.docx]

Supporting information

Nuclear magnetic resonance-based metabolomics approach to evaluate the prevention effect of *Camellia nitidissima Chi* on colitis-associated carcinogenesis

**Ming-Hui Li^1#^, Hong-Zhi Du^2#^ ,Gui-Ju Kong^2^ , Li-Bao Liu^3^, Xin-Xin Li^4^, Sen-Sen Lin^2^, Ai-Qun Jia^5^, Sheng-Tao Yuan^2*^, Li Sun^2*^, Jun-Song Wang^1*^**

^1^Center for Molecular Metabolism, Nanjing University of Science & Technology, Nanjing, P.R. China

^2^Jiangsu Key Laboratory of Drug Screening and Jiangsu Center for Pharmacodynamics Research and Evaluation, China Pharmaceutical University, Nanjing, P.R. China

^3^Department of Cardiothoracic Surgery, The 3rd Affiliated Hospital, Sun Yat-Sen University, Guangzhou, P.R. China

^4^Tasly Research Institute, Tianjin Tasly Holding Group Co. Ltd., Tianjin, P.R. China

^5^School of Environmental and Biological Engineering, Nanjing University of Science & Technology, Nanjing, P.R. China

**^#^ These authors contributed equally to this work.**

*** Correspondence:**Jun-SongWang
wang.junsong@gmail.com
Li Sun
sunli@cpu.edu.cn
Sheng-TaoYuan
cpuYuanst@163.com


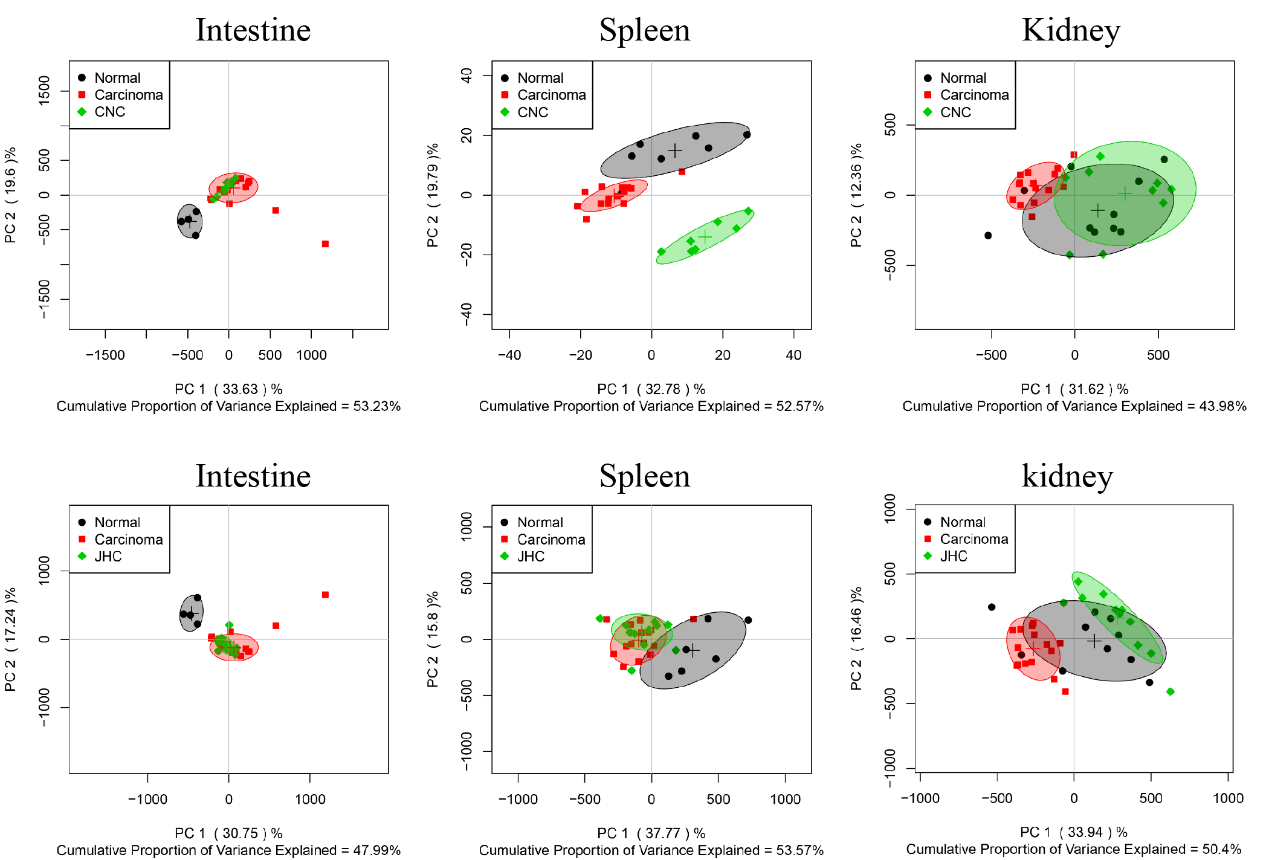


Fig. S1 PCA score plots of control, model and CNC groups as well as control, model and JHC groups for tissues of intestines, spleens and kidneys, respectively.


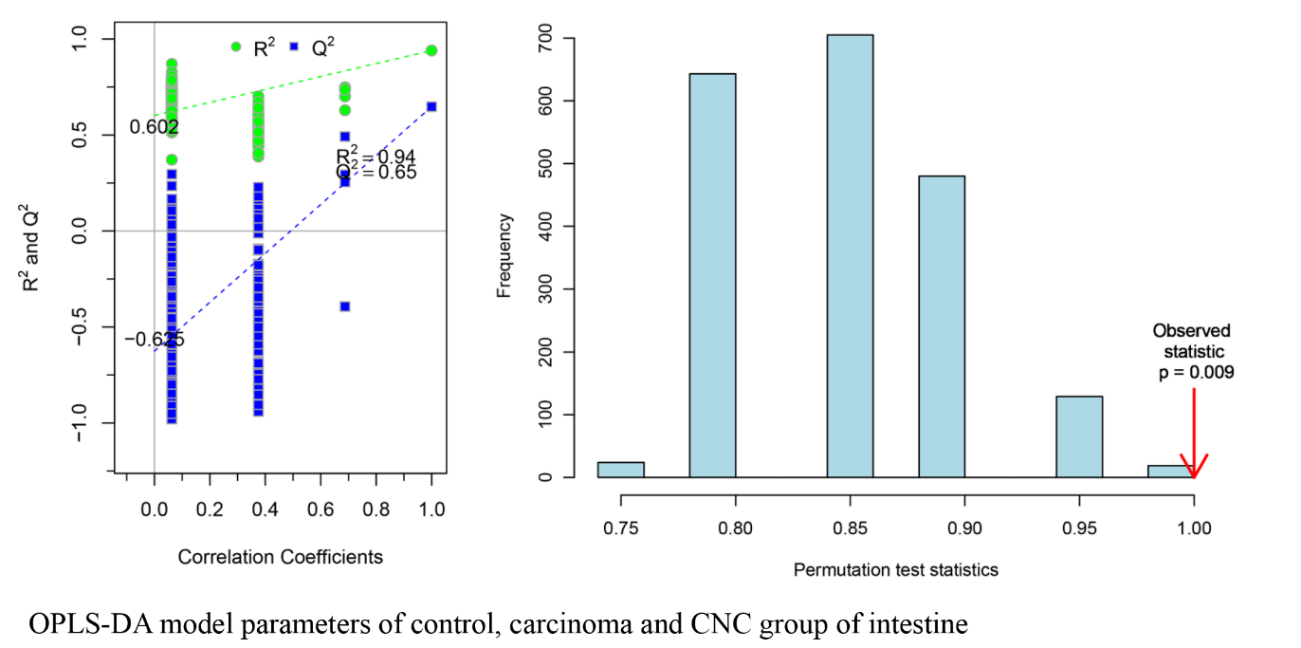


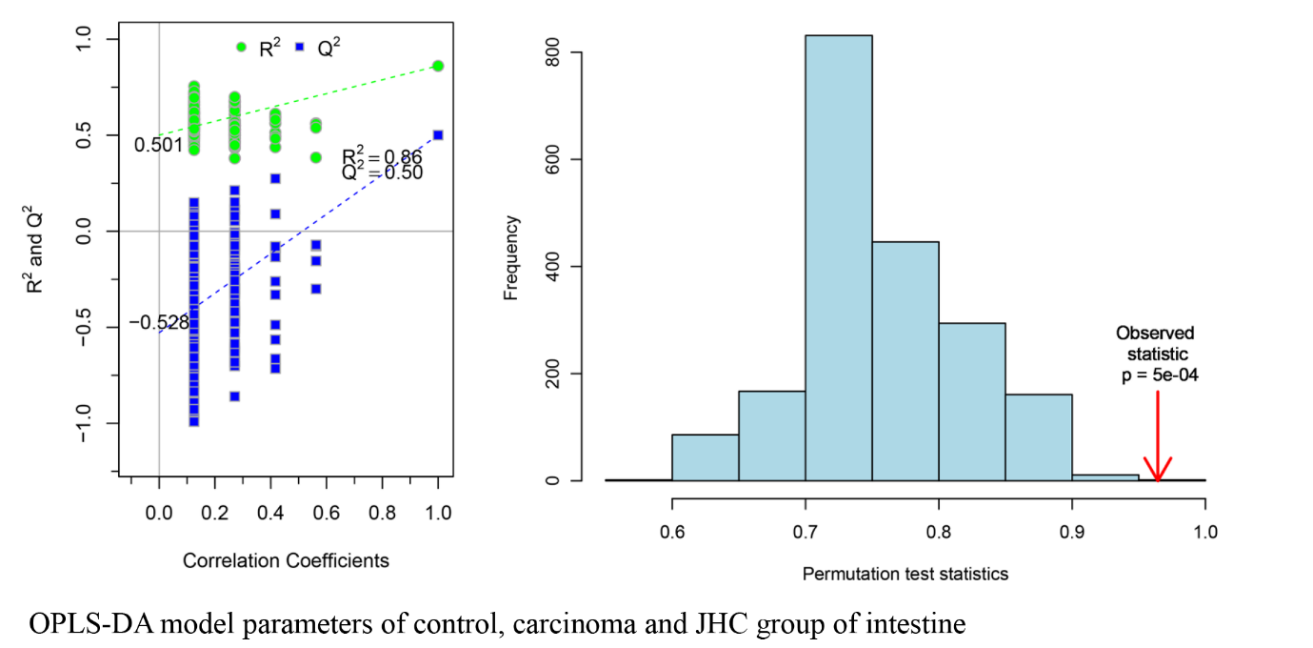


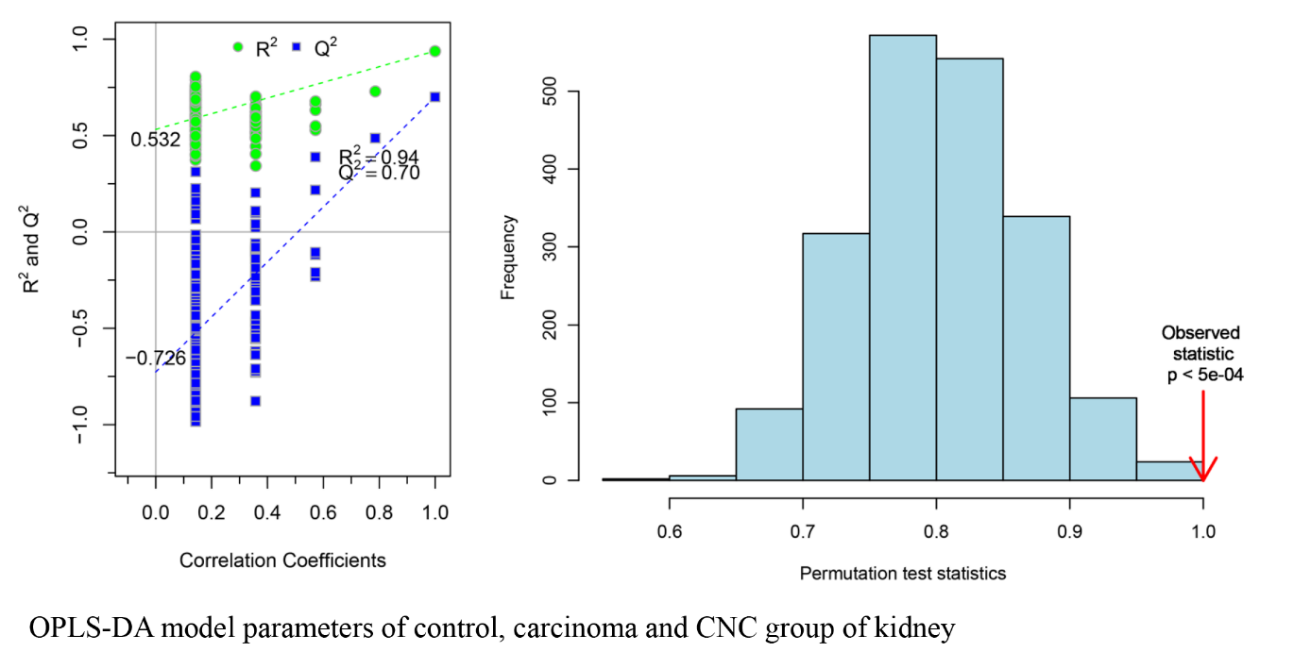


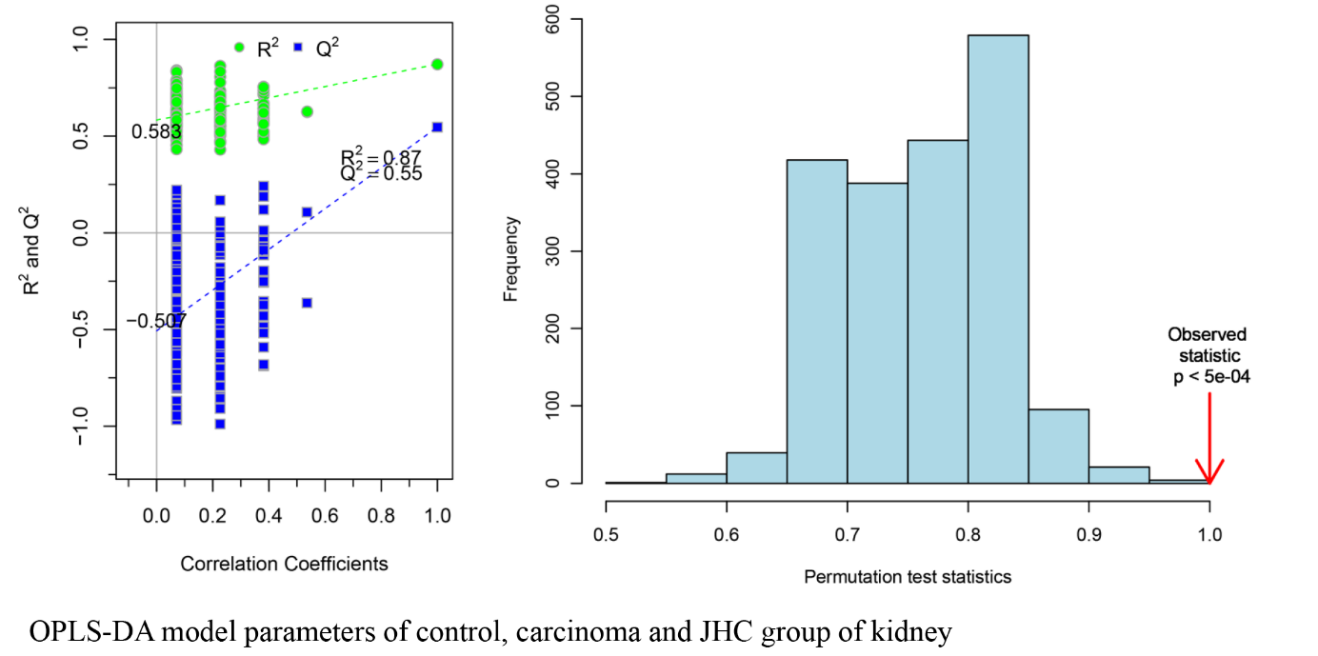


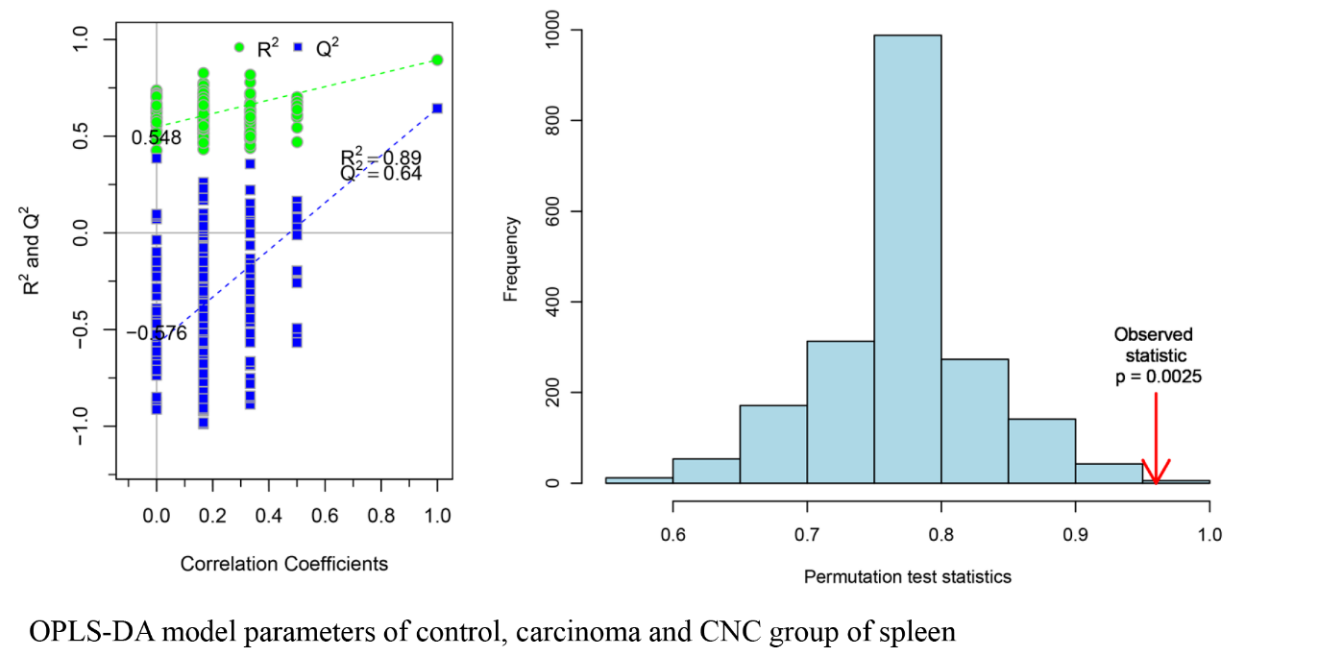


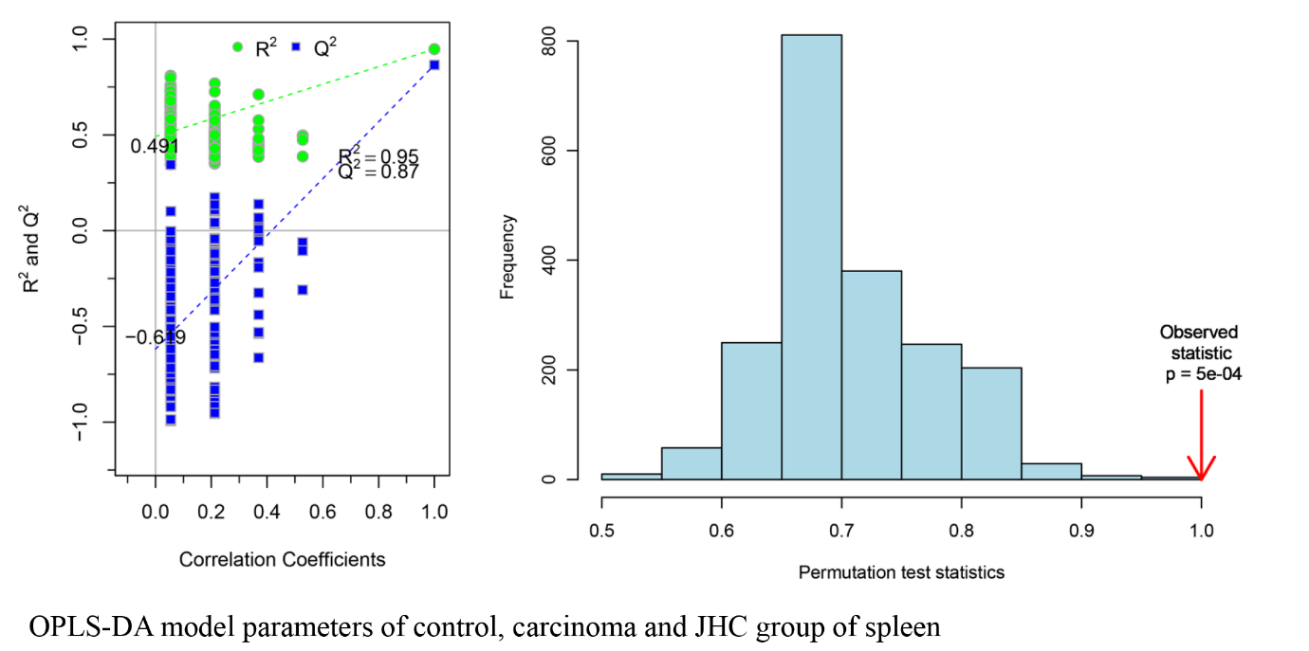


Fig. S2 Scatter plots (left) and histograms (right) for OPLS-DA model validation. Left: Scatter plots of statistical validation obtained by 2000 times permutation test. With R^2^ and Q^2^ values in the vertical axis, the correlation coefficients (between the permuted and true class) in the horizontal axis, and the ordinary least squares (OLS) line for the regression of R^2^ and Q^2^ on the correlation coefficients. Right: Histograms for permutation test scores of various models of tissues. The red arrow indicating the performance based on the original labels, significant for a *p*-value less than 0.05.


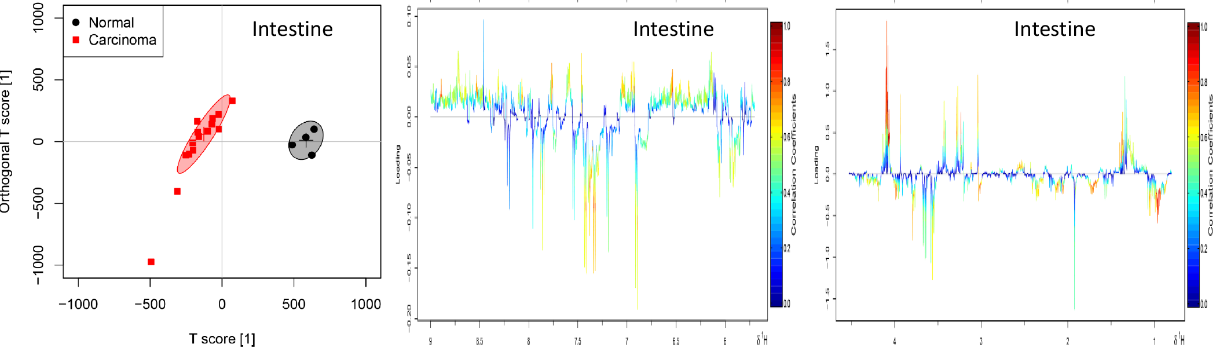


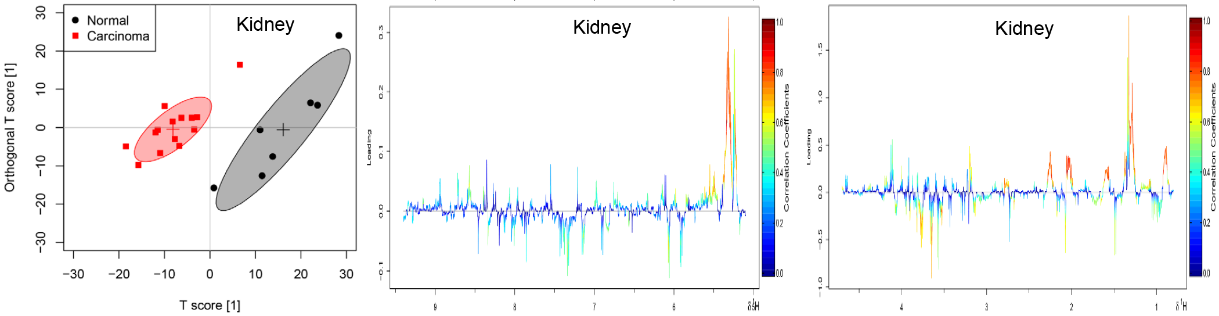


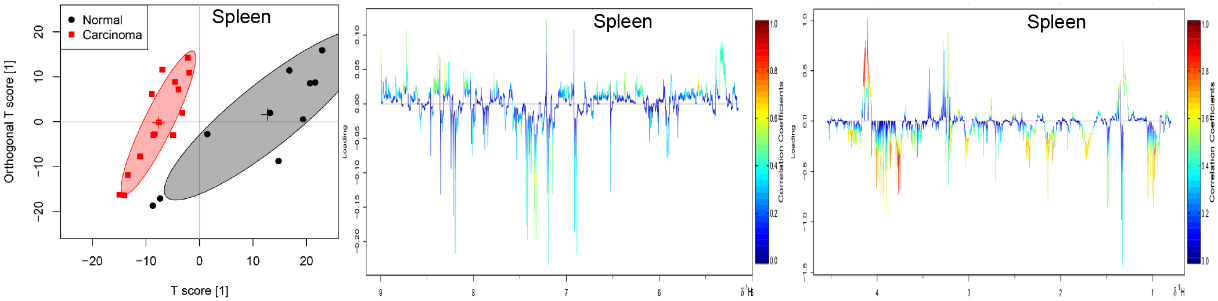

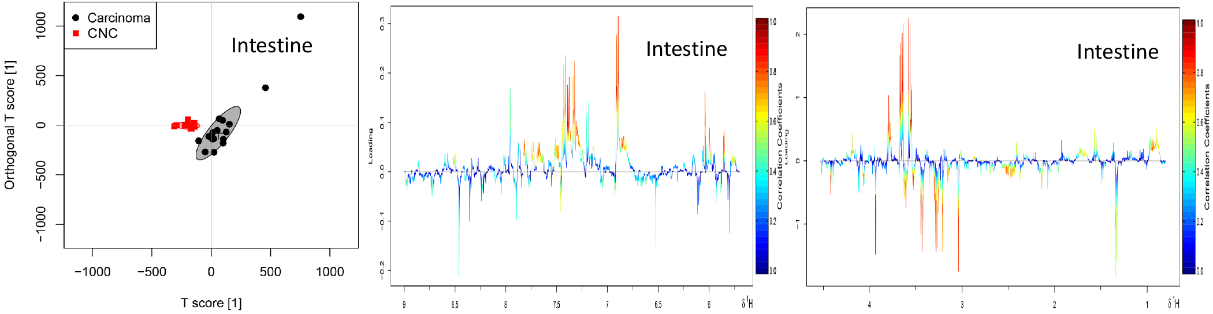

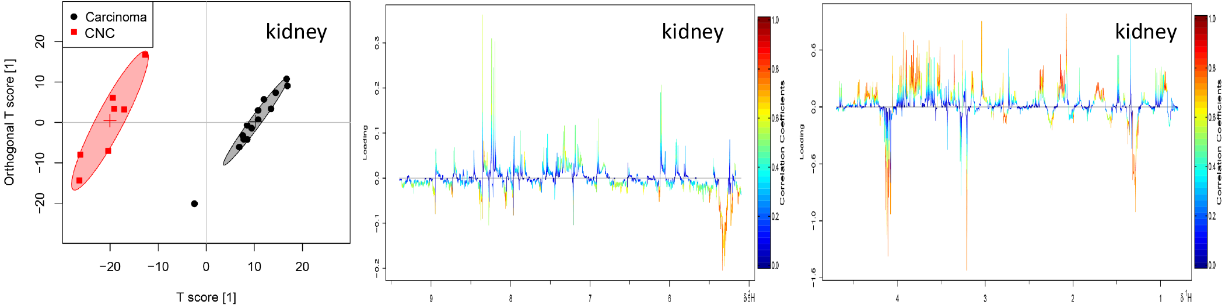

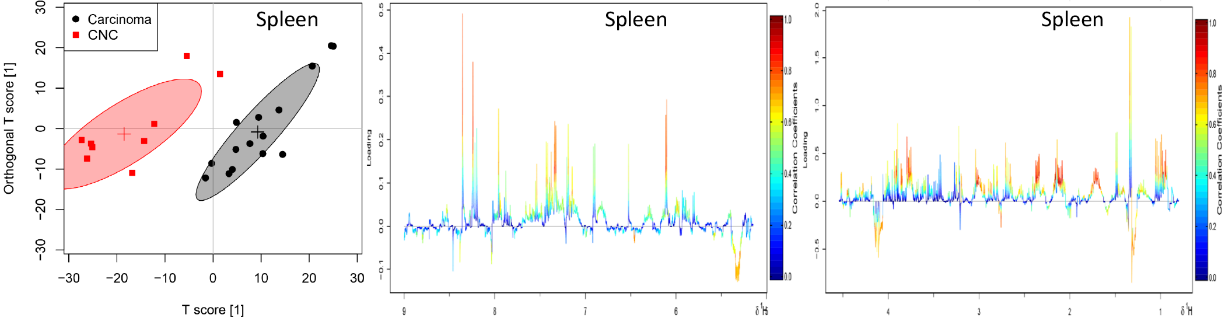


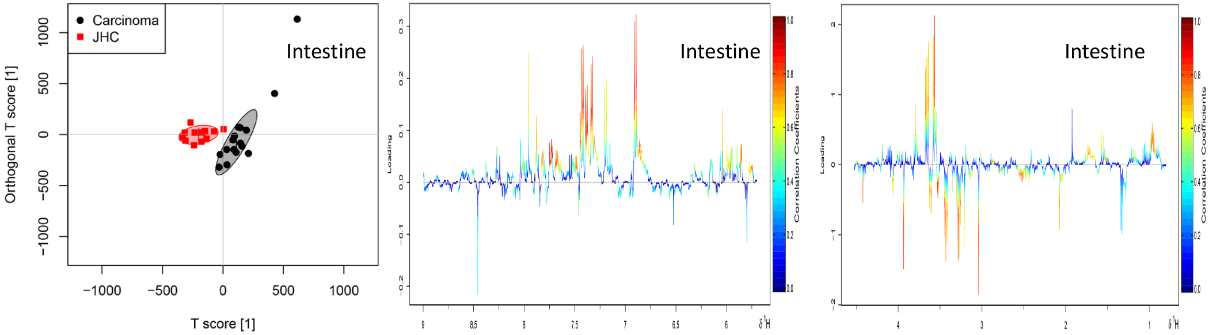

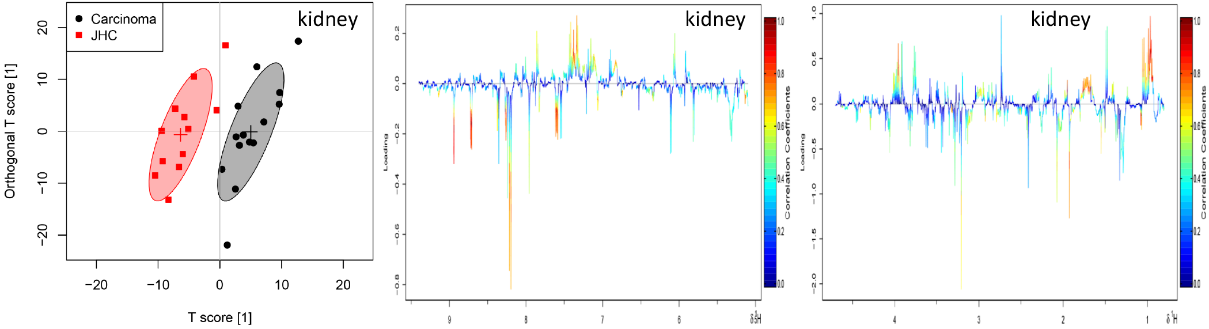


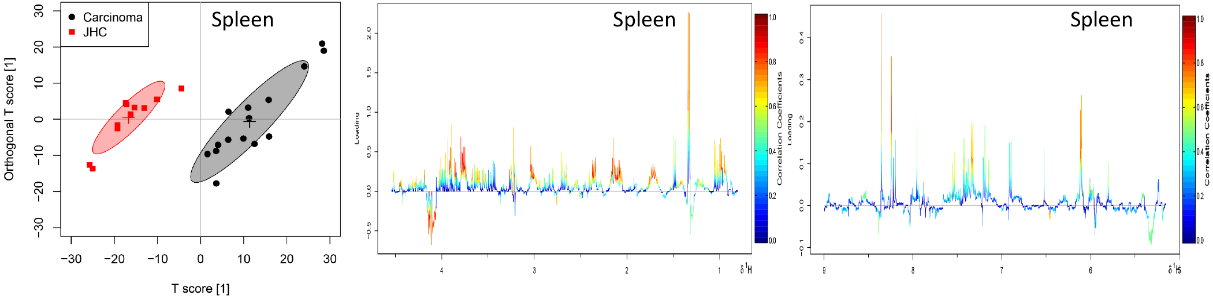


Fig. S3 OPLS-DA models of control *vs* carcinoma, carcinoma *vs* JHC and carcinoma *vs* CNC groups for intestine, kidney and spleen, respectively.
